# Modelling Trial-by-Trial Changes in the Mismatch Negativity

**DOI:** 10.1371/journal.pcbi.1002911

**Published:** 2013-02-21

**Authors:** Falk Lieder, Jean Daunizeau, Marta I. Garrido, Karl J. Friston, Klaas E. Stephan

**Affiliations:** 1 Translational Neuromodeling Unit (TNU), Institute of Biomedical Engineering, University of Zurich & ETH Zurich, Zurich, Switzerland; 2 Laboratory for Social and Neural Systems Research (SNS), Department of Economics, University of Zurich, University Hospital Zurich, Zurich, Switzerland; 3 Wellcome Trust Centre for Neuroimaging, Institute of Neurology, University College London, London, United Kingdom; 4 Brain and Spine Institute (ICM), Hôpital Pitié Salpêtrière, Paris, France; 5 Queensland Brain Institute, The University of Queensland, St Lucia, Australia; Indiana University, United States of America

## Abstract

The mismatch negativity (MMN) is a differential brain response to violations of learned regularities. It has been used to demonstrate that the brain learns the statistical structure of its environment and predicts future sensory inputs. However, the algorithmic nature of these computations and the underlying neurobiological implementation remain controversial. This article introduces a mathematical framework with which competing ideas about the computational quantities indexed by MMN responses can be formalized and tested against single-trial EEG data. This framework was applied to five major theories of the MMN, comparing their ability to explain trial-by-trial changes in MMN amplitude. Three of these theories (predictive coding, model adjustment, and novelty detection) were formalized by linking the MMN to different manifestations of the same computational mechanism: approximate Bayesian inference according to the free-energy principle. We thereby propose a unifying view on three distinct theories of the MMN. The relative plausibility of each theory was assessed against empirical single-trial MMN amplitudes acquired from eight healthy volunteers in a roving oddball experiment. Models based on the free-energy principle provided more plausible explanations of trial-by-trial changes in MMN amplitude than models representing the two more traditional theories (change detection and adaptation). Our results suggest that the MMN reflects approximate Bayesian learning of sensory regularities, and that the MMN-generating process adjusts a probabilistic model of the environment according to prediction errors.

## Author Summary

The ability to predict one's environment is crucial for adaptive and proactive behaviour. It requires learning a mental model that captures the environment's statistical regularities. A process of this sort is thought to be reflected by the mismatch negativity (MMN) potential, a non-invasive electrophysiological measure of the neural response to regularity violation by sensory stimuli. However, the exact computational processes reflected by the MMN remain a matter of debate. We developed a modelling framework in which competing hypotheses about these processes can be objectively compared by their ability to predict single-trial MMN amplitudes. We applied this framework to formalize five major MMN theories and propose a unifying view on three distinct theories which explain the MMN as a reflection of prediction errors, model adjustment, and novelty detection, respectively. We assessed our models of the five theories with EEG data from eight healthy volunteers. Our results are consistent with the idea that the MMN arises from prediction error driven adjustments of a probabilistic mental model of the environment.

## Introduction

A key theme of contemporary neuroscience is the notion that the brain embodies a generative model of the environment, enabling inference on the causes of sensory inputs and predicting future events. This is also known as the “Bayesian brain hypothesis” (for reviews, see [1] and [2]). This framework provides an abstract explanation of adaptive cognition and behaviour, which has been instantiated in schemes like predictive coding and hierarchical Bayesian message passing [3]–[5], or, more recently, the free-energy principle [2].

**Figure 1:**
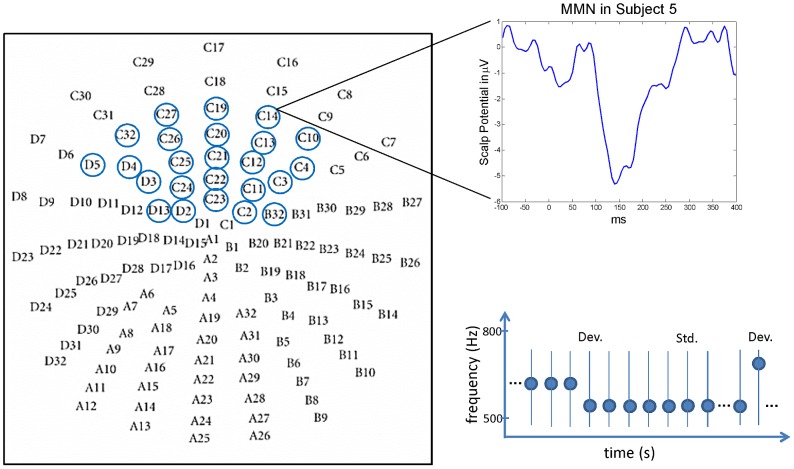
Data acquisition: EEG layout, pre-defined electrodes, sample waveform, and stimulus sequences. The left panel shows the layout of the 128 electrodes of the EEG setup. The blue circles highlight the pre-defined fronto-central electrodes. The upper right panel shows a difference wave containing the MMN. The lower right panel illustrates the structure of the tone sequences presented in the roving oddball experiment. Tones are shown as black disks whose vertical position indicates sound frequency. The first tone presented after a train of tones of a different frequency is called a *deviant* (D).

Experimentally, an important paradigm for testing the implications of these theories in humans is the mismatch negativity (MMN) paradigm [6]. In this paradigm, electrophysiological methods such as electroencephalography (EEG) or magnetoencephalography (MEG) are used to measure event-related “mismatch potentials” in response to violations of expectancy or learned regularities. Traditionally, the MMN (cf. Figure 1) is recorded during auditory oddball experiments or, more recently, during “roving” oddball paradigms. It can be defined operationally by subtracting the event-related potential (ERP) elicited by *standards*, i.e. stimuli that are predicted by an established regularity, from the ERP elicited by *deviants*, i.e. the same stimuli when they violate the regularity. The MMN is usually expressed most strongly at fronto-central electrodes, and its peak latency varies between 100 and 250 milliseconds after deviance onset, depending on the specific paradigm and type of regularity that is violated [7], [8]. Previous EEG and fMRI studies suggest that the MMN originates from temporal generators (A1 and STG) and a prefrontal generator in the inferior frontal gyrus [9], [10].

A major research theme has been the search for models of the neurophysiological and computational processes that underlie the MMN [7], [11], [12]. Such models would contribute to a better understanding of statistical learning in the brain and the prediction of future events. However, the neurocomputational processes that generate the mismatch negativity are still subject to debate [7], [13]–[15]. Over the years, five major hypotheses have been formulated, which we compare in this article:

1. *Change Detection Hypothesis*: The MMN reflects the detection of a local physical change in the sensory input [16], [17].
2. *Adaptation Hypothesis*: The MMN reflects the difference in stimulus-evoked activity between adapted and non-adapted sensory neurons [13], [18].
3. *Model Adjustment Hypothesis*: The auditory cortex maintains a model of the acoustic environment, and stimulus-induced updates of this model are indexed by the MMN [19], [20].
4. *Novelty Detection Hypothesis*: The MMN reflects the degree to which the current event is surprising (novel) [21], [22]. An event is surprising, if its occurrence violates a (probabilistic) prediction. Surprise is different from change: when a change occurs predictably in a given context, its absence will be more surprising than its presence. Surprise is an undirected quantity; this distinguishes it from prediction error (see below).
5. *Prediction Error Hypothesis*: The cortex implements approximate Bayesian inference using predictive coding. The MMN reflects the neural activity encoding the prediction errors that drive this process; i.e., differences between actual and predicted inputs [3], [7]. In contrast to surprise, a prediction error indicates the direction in which the event deviated from the brain's prediction.

So far there has been no objective procedure to conclude which MMN theory is best supported by a given dataset, because most theories of the MMN are of a qualitative nature and do not make quantitative predictions. Furthermore, the inferences that could be drawn were limited by the averaging inherent to standard ERP analysis: this destroys any information about the temporal dynamics of learning. The first goal of this study was to overcome both limitations by providing a modelling framework with which competing MMN theories can be formalized and objectively compared against one another by their capacity to explain single-trial MMN amplitudes. Here, the explanandum was not just the mismatch negativity *per se*, but also how its single-trial amplitude *changes* as the subject learns statistical regularities during the successive presentation of stimuli. The mismatch response to the same stimulus differs depending on the history of all preceding stimuli, and our models should be able to predict these changes. The ensuing modelling of single-trial MMN amplitudes and their progressive changes represents a novel approach, which emphasizes the sensory learning on which the MMN rests. Two related studies using a similar approach recently suggested that single-trial MMN and P300 amplitudes reflect the trial-wise degree of Bayesian and Shannon surprise, respectively [23], [24]. Here, we extend this trial-wise approach and formalize the *processes* postulated by the five MMN theories introduced above in terms of specific process models; these are then subjected to Bayesian model comparison in order to assess how well each of them explains the variability of trial-wise MMN amplitudes. This formulation of detailed and quantitative models representing the 5 major contemporary MMN theories constituted the second goal of this paper. In constructing these models, the third goal was to show that the prediction error, model adjustment, and novelty detection theories of the MMN can be unified. Concretely, we propose that prediction errors, model adjustments and novelty are different manifestations of a common underlying process, namely variational free-energy minimization during perceptual inference and learning [2].

This paper is structured as follows. The *Models and Methods* section describes our roving oddball experiment, data acquisition and pre-processing, the extraction of the single-trial MMN amplitudes used in the subsequent analysis, as well as our modelling framework and its application to formalizing each of five MMN theories by a model family (a set of models with a shared essence). The two final sections present and discuss the results obtained by fitting the ensuing models to empirical MMN responses and applying Bayesian model comparison to assess the relative plausibility of individual models and MMN theories (model families).

## Models and Methods

### Roving paradigm and event related potentials

The empirical data used in this study comprised trial-wise mismatch responses, acquired during a roving oddball experiment with electroencephalography (EEG) from eight healthy subjects in a previously published study [25], [26]. Twelve healthy volunteers (aged 24–34, 4 female) listened passively to a structured sequence of 1600 pure sine tones adapted from [27]. Subjects sat in front of a computer screen and were instructed to ignore the tones and press a button whenever there was a change in the luminance of the fixation cross. The structure of the stimulus sequences is illustrated in Figure 1 (lower right panel). For each subject, the stimulus sequence was structured into approx. 250 trains of a varying number of identical tones, each of which was followed by a train of tones with a different frequency. In other words, the same tone was repeated several times and then changed to a new tone. This lead to two types of events: tone repetition and tone change. The probabilities of trains with zero to ten tone repetitions were 2.5%, 2.5%, 3.75%, 3.75%, 12.5%, 12.5%, 12.5%, 12.5%, 12.5%, 12.5%, and 12.5%. The tone frequencies were 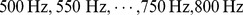, and they occurred with equal probability in a pseudorandom order. Tones lasted for 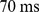 and were presented at a constant stimulus onset asynchrony of 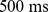 for 15 minutes using headphones.

In this study, we quantified the MMN by subtracting the average of waveforms elicited by the sixth presentation of a tone (the *standard*) from the waveform elicited by its first presentation (the *deviant*). In other words, we compared responses to physically identical stimuli presented in different contexts (i.e. after different stimulus trains). This avoids confounding factors that would have arisen had we used a classical oddball or mismatch negativity paradigm [28] for our single-trial analysis (e.g., differences in physical stimulus properties between standards and deviants and differences in the degree to which the standard was expected [27]).

#### Data acquisition and pre-processing

The data were acquired using a Biosemi EEG setup with 128 electrodes. Data pre-processing was performed with SPM5. Artefact correction was performed by thresholding all channels at 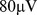. Two subjects were excluded due to artefacts (as in the original study [25]) and two further subjects were excluded due to a low signal-to-noise ratio or undetectable MMN (as in [26]), leaving eight subjects for the final analysis. We selected a pre-defined set of fronto-central electrodes based on studies that have differentiated between the temporal and the frontal MMN subcomponent [29]–[33] and analysed the potentials at these electrodes and all electrodes located between them. Figure 1 (left panel) shows the spatial layout of these electrodes. Subject-specific subsets of the preselected electrodes were created by excluding those electrodes where the expected mismatch potential could not be detected in the subject's average difference wave. The detection of MMN was performed by t-tests comparing the mean potential within the time-window of the MMN with the mean potentials in two surrounding time windows (before and after). The critical value of each test was chosen according to the Šidák correction such that a family-wise error, i.e. erroneously selecting at least one channel, would occur with a probability of less than 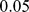.

#### Estimation of single-trial MMN amplitudes

The data feature that we modelled is the sequence of single-trial MMN amplitudes that has one element for each deviant trial. Each deviant trial is characterised by the tone (frequency) and the length of the preceding train of tone repetitions.

For each subject and each deviant trial the MMN amplitude was estimated by applying the procedure of Mars et al. [24] separately to all selected channels. In short, this involved:

1. For each deviant trial, subtract the “standard ERP” of the presented tone (average response across all trials presenting the tone for the sixth time in a row) from the EEG signal recorded in that trial. This isolates the deviance-specific potential.
2. For each deviance-specific potential, subtract the average potential in the 100 ms preceding the deviant presentation from the ensuing response (baseline correction).
3. Estimate each subject's MMN peak latency by the minimum point of his/her average difference wave (average of deviance-specific potentials across deviant trials and selected frontal electrodes) between 100 and 200 ms after stimulus onset [25].
4. Estimate each subject's trial-wise MMN amplitudes by averaging his/her deviance-specific potentials over a 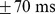 time window centered at his/her MMN peak latency. The window's width (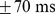) was chosen to match the duration of the MMN.

### A framework for modelling single-trial responses

This section introduces our modelling framework for single-trial responses. In terms of notation, we denote vectors by lower case bold letters, matrices by upper case bold letters, and scalars and functions by lower case italics (except for variables like the free-energy 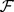 for which there are notational conventions in the literature). Vector and matrix elements can be scalars, vectors, or matrices, and they are referred to via subscripts (e.g., 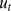 denotes the t^th^ element of vector 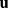, and 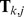 denotes the j^th^ element of the k^th^ row of matrix 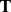).

Models of single-trial responses can be cast in a general dynamic state-space framework that models the measurements 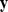 as manifestations of internal states 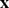 which cannot be observed directly. The internal states evolve according to an evolution function 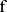 mapping an internal state and some sensory input 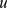 to the ensuing state. The internal states 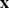 generate neurophysiological signals in response to sensory input according to a response function 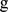. These are scaled and combined according to a linear observation model with regression coefficients 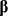 and corrupted by Gaussian measurement noise 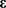. Both the evolution function and the response function may depend on parameters 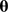 and have the following general form:
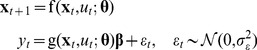
(1)Together with the prior density 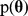, the evolution function and the response function define a generative model of the measurements:
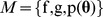
(2)This framework is based on [34] and enables inferences about (hidden) computational processes and representations from neurophysiological measurements. It is particularly powerful in conjunction with model comparison methods such as random-effects Bayesian model selection [35] and model space partitioning (i.e., inference on model families [36]). Given competing models of learning and inference, Bayesian model inversion and comparison can be used to infer the nature of the underlying process and its relationship to the measured responses. The resulting posterior model probabilities assess each model's relative explanatory power in a way that balances fit and complexity such that the comparison between any two models is valid irrespective of their relative complexity.

### Computational models of the mismatch negativity

We applied the framework introduced in the previous section to formalize five competing theories of the MMN by formulating thirteen models (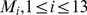) of measured trial-wise MMN amplitudes 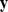 elicited by tone sequences 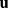. Each of the five theories summarized in the introduction (predictive coding, novelty detection, model adjustment, change detection, and adaptation) explains the MMN as originating from a particular process 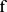 operating on some neural state or cognitive representation 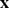. We modelled these processes and representations as well as the resulting neural responses 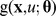 which we interpret as local field potentials. Since the EEG signal is a linear mixture of local field potentials, we use a general linear model to map predicted neuronal activity to MMN amplitude; this is expressed by Eq. (3) where 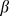 are the unknown regression coefficients, and the trial-wise values of 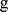 define the design matrix:
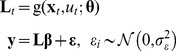
(3)Note that this is an equation for a single electrode (we generalize it to multiple electrodes in Eq. (13)).

**Figure 2:**
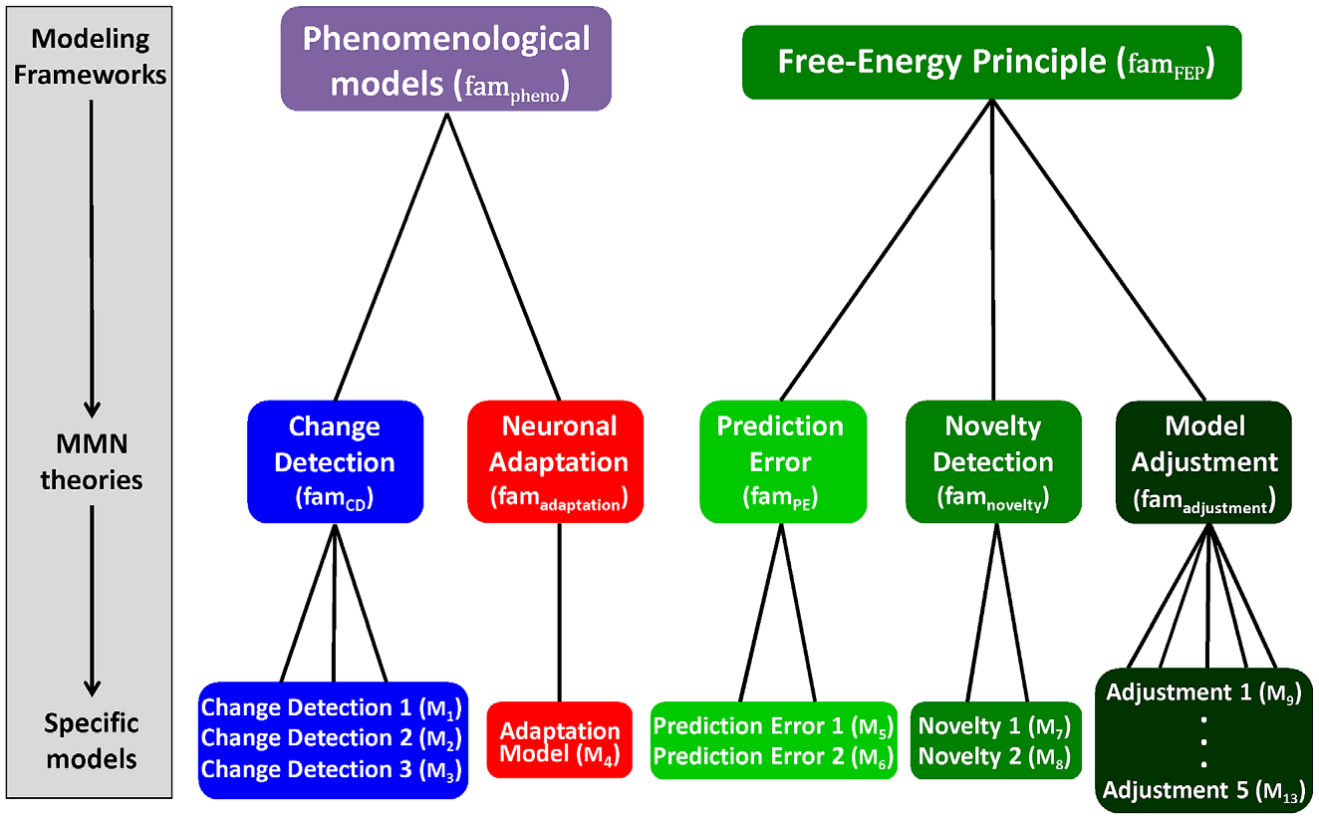
Hierarchical structure of the model space: models, theories, and frameworks. The MMN models developed in this article can be organized into a tree structure. The leaves at the bottom of the tree represent individual models of trial-wise MMN amplitudes, and the nodes above represent sets of models (model families). The nodes at the third level represent modelling frameworks. Three theories (the prediction error hypothesis, the novelty detection hypothesis, and model adjustment hypothesis) were formalized under the framework of the free-energy principle (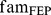). This framework explicitly models information processing, which makes it fundamentally different from phenomenological explanations (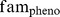), such as change detection and adaptation models.

The 13 models 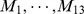 are derived in detail below. After formalizing two traditional phenomenological MMN theories (the change detection hypothesis and the adaptation hypothesis), we formalize three current theories of the MMN using Bayesian information processing models based on the free-energy principle. These models assume that the brain represents probabilistic beliefs about its environment whose evolution approximates Bayes optimal learning and perception according to the free-energy principle [37]. The predictive coding, the model adjustment, and the novelty detection theories were formalized by extending this core assumption by response models 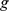 of different neural sub-processes of the belief updates prescribed by the free-energy principle. Overall, our model space is structured hierarchically, as shown in Figure 2. First, our 13 models can be grouped into five model families that correspond to the five MMN theories introduced above: change detection (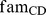), adaptation (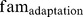), prediction error (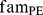), novelty (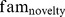), and model adjustment (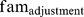). The models within each family assume the same internal representation and the same evolution function, but differ in their response functions. Second, these model families can be grouped into two super-families: phenomenological models (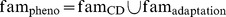) and information processing models (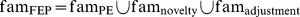). The latter are formulated within a Meta-Bayesian framework [34] and build upon the free-energy principle [37]. Table 1 summarizes all computational models, and the notation used to describe them is summarized in Table 2.

**Table 1:** This table lists the response models of our 13 computational models of trial-wise MMN amplitudes.

Model Name	Estimates generating LFPs	Description
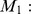 Change Detection 1	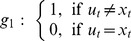	categorical response: change or no change
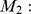 Change Detection 2	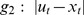	absolute change in log-frequency
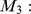 Change Detection 3	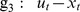	change in log-frequency
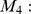 Adaptation 1	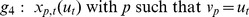	response of adapted neurons selective to the deviant
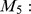 FEP, Prediction Error 1	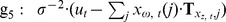	precision weighted prediction error (wrt. sensory inputs)
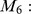 FEP, Prediction Error 2	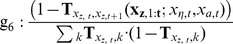	precision weighted prediction error (wrt. tone category)
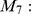 FEP, Novelty 1	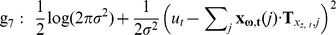	surprise about the sensory input
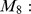 FEP, Novelty 2	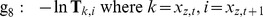	surprise about tone category
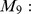 FEP, Model adjustment 1	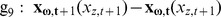	change in the category's mean frequency
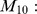 FEP, Model adjustment 2	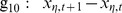	change in expected sequence length
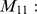 FEP, Model adjustment 3	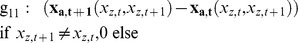	(absolute value of) change in conditional transition prob.
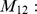 FEP, Model adjustment 4	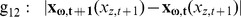	absolute value of change in the category's mean freq.
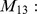 FEP, Model adjustment 5	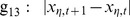	absolute value of change in expected sequence length

The equations specify the trial-wise predictor variables 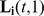. The third column explains the hypothesis formalized by each model. The mathematical notation is explained in Table 2. In both tables the elements of vectors and matrices are sometimes referred to via indices in parentheses such as in 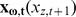 which denotes the element of the vector 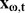 whose index is 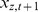. For brevity the response functions 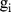 are written in terms of 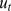, 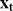, 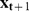, and 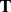. This is consistent with the general state-space framework (Eq. (1)), because 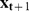 and 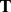 are fully determined by 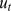 and 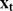.

**Table 2:** Explanation of the variables in our computational models of trial-wise MMN amplitudes.

Variable	Explanation
**Inputs**	
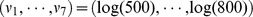	log-frequencies (Hz) of tones presented in the roving oddball exp.
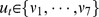	sensory input on trial t
**Output**	
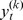	MMN amplitude evoked by the t^th^ deviant at the k^th^ electrode
**Modelling Framework**	
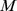	model of trial-wise MMN amplitudes
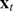	internal state in trial 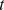
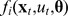	evolution function mapping the current state and the sensory input to the next state
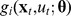	response function of model 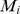 , maps internal state and sensory input to neural response
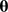	subject-specific parameters of the evolution and response functions
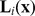	predictors of local field potentials implied by internal states 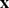 and response function 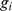
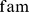	model family: set of models with a common characteristic
**Internal States of Change Detection Models**	
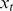	represents input of current and previous trial (memory trace)
**Internal States of Adaption Model**	
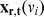	responsiveness of neurons selective to log-frequency *v_i_* in trial *t*
**Internal States of Bayesian Observer (FEP Models)**	
M	probabilistic mental model of tone sequences
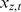	belief about category of the previous tone
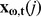	belief about characteristic log-frequency of the j-th tone category
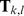	belief about transition probability from hidden state k to hidden state l
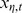	belief about average sequence length
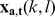	belief about the probability of a transition from category k to category l given that a change occurs
**Parameters (** 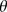 **) of the Change Detection Models**	
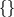	This model family has no free parameters.
**Parameters (** 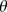 **) of the Adaptation Model**	
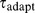	time constant of the adaptation process
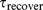	time constant of the recovery from adaptation
**Parameters (** 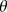 **) of the Bayesian Observer (FEP Models)**	
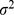	perceptual uncertainty
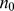	strength of prior beliefs (number of virtual tone sequences observed prior to the experiment)
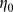	prior expectation of tone sequence lengths

#### Change detection hypothesis (Models M_1_-M_3_)

A classical interpretation of the MMN is the *change detection hypothesis*, which assumes that the MMN indexes local physical changes in the sensory input [16], [17]. This hypothesis comes in several flavours, each of which leads to different quantitative predictions.

1. The MMN indexes only whether or not a change has occurred.
2. The MMN indexes the absolute value of the change in a physical property of the sensory input (i.e., unsigned change).
3. The MMN indexes the difference in a physical property between the deviant and its predecessor (i.e., signed change).

Here, the relevant physical stimulus property is the log-frequency of a pure sine tone. In our framework, the general notion of change detection can be formalized by assuming a one-dimensional internal representation 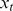 of previous sensory input:
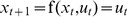
(4)This internal representation 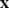 and evolution function 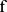 are shared by all three variants of the change detection hypothesis summarised above. Their divergent interpretations simply rest on what trial-wise MMN amplitudes depend on; this was expressed by three different response functions:
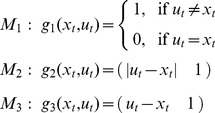
(5)Notably, 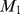 can be considered a null model, since, in contrast to all other models in this paper, it postulates that there is no trial-by-trial variation in MMN amplitude. It predicts the same MMN amplitude for all modelled trials (and therefore does not include an additional constant, cf. Eq. (5)). The second and third model assume that MMN amplitude increases linearly with the change in log-frequency (cf. [22]), but differ with regard to whether or not this effect depends on the sign of the difference. Altogether, these three models constitute the “change detection” family 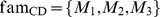 (see Figure 2).

#### Adaptation hypothesis (Model M_4_)

Neural adaptation is the process due to which the neural response to a stimulus or feature decreases with its repeated or prolonged presentation. According to the *adaptation hypothesis*, the MMN elicited by a change in sound frequency reflects the difference in the responsiveness of adapted and non-adapted frequency-specific neurons in auditory cortex [12]. For instance, it has been suggested that the MMN results from a delay and an attenuation of the N1 component due to neuronal adaptation [18]. Invasive recording studies have shown that the firing rate of neurons selective for the standard frequency decreases monotonically with the number of standard repetitions [38], [39], and that this adaptation is expressed at multiple time scales: from hundreds of milliseconds to tens of seconds. These adaptation effects could result from mechanisms at the level of single neurons and synapses; e.g. synaptic depression [40] or slow after-hyperpolarizing potassium currents [41]. Alternative explanations include network mechanisms such as cascades of depressing synapses [42] or predictive coding; where adaptation is mediated by local connections that control the gain of error units [26].

Here, we adopted a phenomenological description of adaptation that is agnostic to the exact underlying mechanism. We modelled seven populations of frequency-selective neurons, each of which is responsive to exactly one of the seven log-frequencies 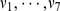 presented in our roving oddball experiment. The internal states are therefore represented by a seven-dimensional vector 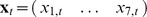 encoding the current responsiveness of each neural population to its preferred stimulus frequency. Following [38], we model the responsiveness of each frequency-specific population using two exponential processes. Each population's responsiveness decays and recovers exponentially with the number of presentations of its preferred frequency and non-preferred frequencies, respectively. This is captured by the adaptation model's evolution function
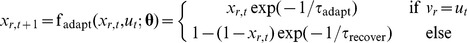
(6)where the free parameters 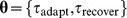 capture the time scales at which the adaptation and the recovery process operate and are allowed to vary across subjects. These parameters were assigned uniform prior distributions covering the full range of plausible values reported in [38], i.e. 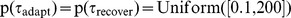.

This model predicts that the MMN amplitude is proportional to the responsiveness of the stimulus-driven neuronal population. Therefore, the response function simply reads out the appropriate state value and combines it with a constant:
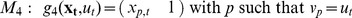
(7)In summary, this generative model 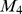 explains trial-wise MMN amplitudes in terms of two processes: adaptation and recovery from adaptation. This model constitutes the “adaptation” model family 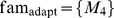 of our model space (see Figure 2).

#### Predictive coding, model adjustment, and novelty detection

**Figure 3:**
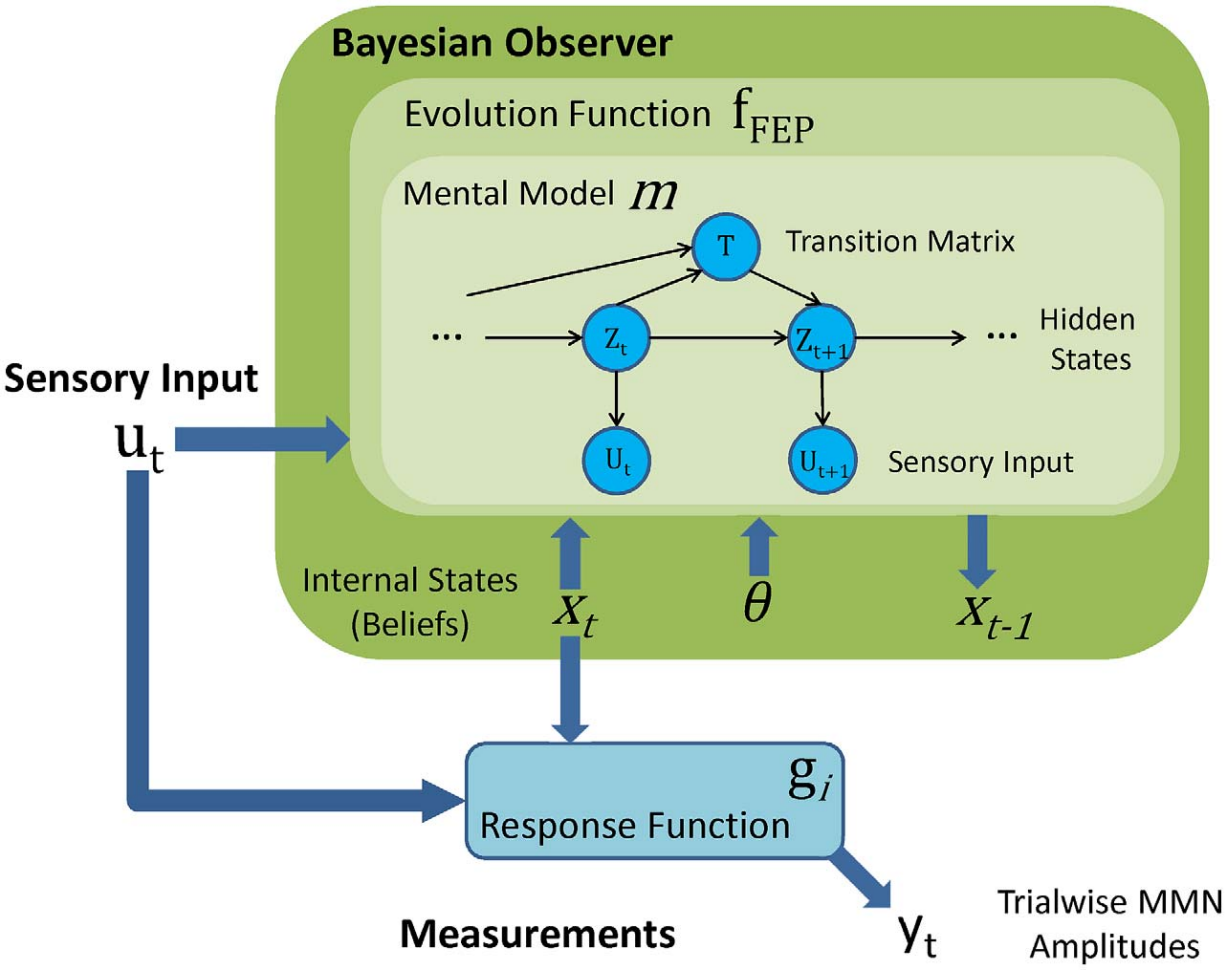
Structure of free-energy based models of the MMN. Our free-energy models of trial-wise MMN amplitudes (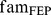 in Figure 2) are cast within the general dynamic state-space framework formulated in Equation (1). In contrast to the phenomenological models, the internal states (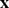) represent probabilistic beliefs about the environment and evolve according to approximate Bayesian inference by free-energy minimization (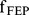). All of these models share the Bayesian observer defined by the evolution function 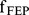 and the probabilistic mental model 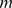, but differ in their response functions 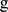. The graph in the innermost box shows the mental model 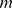 as a probabilistic graphical model (with arrows indicating conditional dependencies). The random variables in circles are sensory inputs (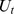), tone categories (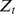), and transition probabilities (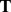). This mental model determines how subjects perceive, learn about and predict tone sequences. Please see Table 2 for an explanation of the mathematical notation.

*Predictive coding, model adjustment*, and *novelty detection* are formalized by models based on the free-energy principle (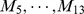). These models explain the MMN as an electrophysiological manifestation of the neural mechanisms that approximate Bayes-optimal perception and learning of sensory regularities. Figure 3 illustrates that these models are structured into two components: a Bayesian observer and a response function. This instantiates our general dynamic state-space framework: The internal states 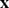 represent the Bayesian observer's probabilistic beliefs, and the response functions map belief updates to neural responses. The Bayesian observer is shared by all information processing models; it is their response functions 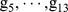 (summarized in Table 1) that differentiate them into models of predictive coding, novelty detection, or model adjustment. As shown in Figure 3, the beliefs of the Bayesian observer evolve according to an evolution function that depends on the observer's mental model. The following two subsections introduce this mental model and the evolution function respectively, and the third subsection introduces the response functions. The notation used to describe the Bayesian observer model is summarized in Table 2.

#### The Bayesian observer's mental model of tone sequences

We approximate the subjects' mental model of tone sequences by an extension of the hidden Markov model; see Figure 3. This model captures the general principle that the states of the environment (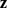) are unobservable and have to be inferred from sensory inputs (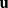). Concretely, on each trial of the roving oddball experiment, the auditory cortex receives sensory input 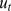 that can be thought of as the sound frequency represented on a logarithmic scale [43]–[45] by neural activity in the auditory thalamus (medial geniculate nucleus, MGN), a key relay station of the ascending auditory pathway which provides input to the primary auditory cortex [46].

In our model the hidden environmental state 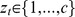 represents the category of the *t* ^th^ tone, e.g. which musical note it instantiates (note that 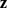 is an environmental event and thus a cause of sensory input; whereas 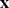 is an internal state of the brain which we will assume to encode the sufficient statistics of the approximate posterior 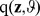; see below). Each tone category has a characteristic log-frequency 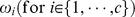, but sounds sampled from it deviate randomly. We assume that the subjects' initial tone categories approximately correspond to musical notes, because for pure tones subjects' auditory representations are likely to be shaped by musical experience, and pitch perception becomes increasingly logarithmic for frequencies above 500 Hz [47]. Since the tones presented in the experiment range from 500 to 800 Hz, we simulated categories corresponding to the musical notes from B4 (493.88 Hz) to A^b^5 (830.61 Hz). As a result, the mental model contains 10 tone categories (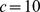), and the learner updates its estimates of their characteristic frequencies based on sensory input.

While the relationship between the perceived frequency (pitch) of complex sounds and their physical properties is complicated [48], the log-frequency of pure sine tones is accurately encoded by the cochlea [49]. Thus, for pure sine tones the log-frequency representation of sensory data can be plausibly modelled with:
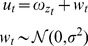
(8)where 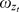 is the characteristic log-frequency of the note presented on trial 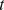 and 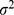 is the variance of the MGN's representation of tone's log-frequency. It corresponds to the observer's perceptual uncertainty and was assumed to be constant and known to the observer.

Furthermore, the temporal structure of the hidden sequence is represented by the transition matrix 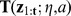 that captures the distribution of the number of tone repetitions irrespective of tone identity, and that certain transitions are more likely than others. The former was achieved by extending the hidden Markov model [50] such that the transition matrix can depend on the history of the hidden states; see Section 1 in Text S1 for details. This extension was motivated by previous MMN studies showing that the number of standard repetitions is an important factor in modulating the amplitude of the MMN [27], [51]–[54]. In brief, the transition matrix depends directly on how often the current tone has been repeated, as well as on the expected number of tone repetitions (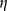) and the conditional probabilities of the next tone given the current tone and given that a change occurs (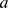). In summary, we assume that the mental model 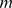 is defined by the following set of assumptions about the observations 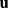, hidden states 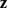 and parameters 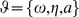:
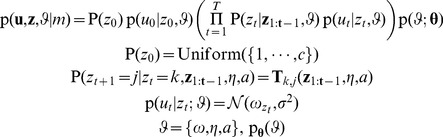
(9)Here, 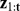 denotes the sequence of hidden states from trial 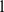 to trial 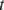. The structure of the transition matrix, its dependence on the history of hidden states, as well as the model parameters and their priors are described in detail in Section 1 of Text S1. Note that we do not make strong assumptions about the prior knowledge each subject brings to the experiment or their perceptual uncertainty. Instead, we infer each subject's prior beliefs 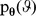 and perceptual uncertainty 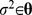 individually by estimating the hyperparameters 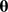
from their data; for details see Section 3 in Text S1. Importantly, the hyperparameters are not properties of the environment learned by the observer, but properties of the observer that must be inferred by the experimenter [34].

#### Evolution function of the Bayesian observer's beliefs

This subsection derives the evolution function 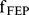 of the Bayesian observer's beliefs from the free-energy principle (FEP). The free-energy principle goes back to Helmholtz's idea that perception is unconscious inference about the state of the world [55]. More recently this idea has been formalized in terms of Bayesian inference. The Bayesian brain hypothesis maintains that the brain computes a probability distribution over the potential causes 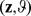 of its sensory inputs 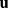 by inverting a mental model 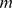 of how its sensory inputs are generated [2], [3], [56]–[59]. The hidden causes comprise the hidden environmental states 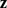 and a set of parameters 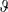 that describe their effects (i.e., how they influence each other and how they cause sensory inputs). The normative solution to this problem is given by Bayes theorem: 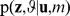. However, evaluating Bayes theorem is intractable for all but the simplest problems. Thus the brain has to use a more efficient but potentially less accurate inference mechanism. According to the free-energy principle, this mechanism optimizes sufficient statistics of a parametric approximation 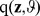 to the posterior density by neural dynamics that minimize the free-energy 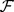 [2], [37], [60]. The free-energy 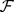 can be expressed as the surprise (about the joint occurrence of the sensory inputs 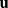, hidden states 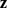 and parameters 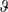) that is expected under an approximate posterior density 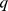, minus the entropy of 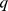 [61]:
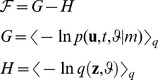
(10)This free-energy framework, which has been used by several recent studies examining learning and inference in the brain [2], [61]–[63], derives from a variational Bayesian [64] perspective on how optimal Bayesian inference could be approximated efficiently. In the following, we use this framework for motivating three families of Bayesian information processing models, in which the internal states 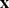 encode the sufficient statistics of the approximate posterior 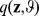. For stimuli that are well known and presented at a very high signal-to-noise ratio, as the sine tones in our case, it is reasonable to assume that the brain encodes these approximate posterior beliefs with point estimates. Thus 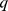 is a delta-distribution and its sufficient statistics are its expectations (which are also the coordinates of its peak), i.e. 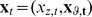 where the first subscript denotes the variable or parameter, and the second subscript denotes the trial that the observer entered with this belief. In other words, 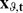 corresponds to prior belief in trial 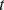 about the parameters of the mental model 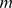, and 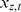 represents the brain's belief (expectation) about the category of the tone presented in trial 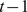. The approximation of posterior beliefs with delta distributions reduces the free-energy to the expected internal energy 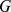:
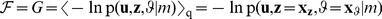
(11)Minimizing free-energy with respect to the sufficient statistics 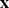 of the approximation 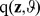 accomplishes both perception (inference on the hidden environmental states 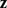) and learning (inference on the parameters 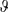). Under the free-energy principle, the temporal evolution function (Eq. (12)) of the observer's beliefs follows directly from the mental model (Eq. (9)) of how sensory inputs are generated; the result is a deterministic function of the current state 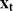 and the sensory input 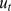:
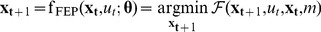
(12)Here, 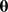 is a set of three hyperparameters that capture interindividual differences in the mental model 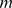 (see Table 2 and Section 3 in Text S1). The evolution function in Eq. 12 is the common core of all nine free-energy models of the MMN (models *M*_5_—*M*_13_ in Figure 2). It derives from a variational scheme that relates free-energy minimization to maximum-a-posteriori inference. It is explained in detail in Section 2 in Text S1, where we have made an effort to link this scheme to putative neurobiological mechanisms (Section 6 in Text S1).

To compute the temporal evolution of the internal states predicted by our free-energy models, the evolution function was iteratively applied to the known sequence of log-frequencies presented in the empirical study. This provides a succession of posterior beliefs that are encoded by neuronal activity and give rise to trial-wise MMN responses. To specify this mapping between posterior beliefs and MMN amplitudes, we now turn to the response models (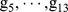).

#### Response functions: From posterior beliefs to the MMN amplitudes

After the preceding sections have described the Bayesian observer, this section describes the response functions specifying how its internal states manifest in measured MMN amplitudes. In the present MMN literature, there are three major hypotheses which can be understood as special cases of the free-energy framework in Figure 3. These hypotheses differ in which particular aspect of sensory learning and perception they postulate to be reflected by the MMN. In our framework, these competing views can be expressed by three classes of response models 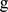 linking the MMN to different neural sub-computations of the belief updates prescribed by the free-energy principle. These response models are briefly summarized here; technical details can be found in Tables 1 and 2, as well as in Section 4 in Text S1.

1. The **p**r**ediction error models** assume that the MMN reflects the activity of neurons encoding precision weighted prediction errors on sensory inputs and hidden states. Roughly speaking, prediction errors are the difference between what is observed and what was predicted from previous experience according to the probabilistic mental model 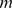. These models appeal to predictive coding [5] formulations of free energy minimization that rest upon hierarchical message passing between representational and prediction error units . Notably, the MMN may be sensitive to prediction errors on sensory inputs, or to prediction errors on hidden states. Each possibility is formalized by a response model (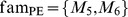; see Table 1).
2. The **novelty detection models** assume that the MMN reflects neuronal activity encoding surprisal (also known as “self-information” or “Shannon surprise”) with respect to the conditional probability distributions describing the observer's beliefs. Unlike prediction error, surprisal is an unsigned quantity, corresponding to the negative logarithm of the conditional probability of sensory inputs given expectations about hidden states (or of hidden states given expectations about model parameters). Because the mental model assumes additive Gaussian noise, the conditional surprise about a stimulus is determined by the precision weighted squared prediction error on the stimulus (equivalently for hidden states). This provides a tractable approximation to the Shannon surprise with respect to the prior predictive density over sensory inputs (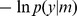) – which, critically, is a formal measure of novelty. This class of response models is thus compatible with hypotheses according to which the MMN indexes an automatic *novelty detection* process [21], [22]. While the first novelty detection model links the MMN to the novelty of sensory inputs, the second novelty detection model links the MMN to the novelty of hidden temporal structure (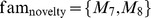; see Table 1).
3. The **“model adjustment” models** assume that trial-wise MMN amplitudes reflect adjustments of the parameters of the probabilistic mental model 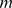; this is a formalization of the *model adjustment hypothesis* [19]. MMN amplitudes could reflect adjustments of different parameters (i.e., the categories' mean frequencies, the expected sequence length, and the conditional transition probabilities) and in different ways (i.e., sensitive or insensitive to the sign of the adjustment). This implies a factorial structure of 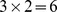 response models. Section 4 in Text S1 provides details and explains why two of these models are redundant, thus resulting in 5 response models for this family (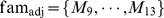; see Table 1).

This completes the formulation of 13 computational models of trial-by-trial changes in MMN amplitude distributed over five model families (see Figure 2). We now proceed to describing family-level Bayesian model selection [36] for evaluating the relative plausibility of the five hypotheses (model families). Importantly, this model comparison at the family levels is less dependent on details of the individual models and thus integrates out uncertainty about how each hypothesis should be formalized exactly.

### Bayesian model selection

Above, we have derived 13 different models predicting the trial-wise MMN amplitudes during our roving oddball experiment. These models differ in numerous ways, conceptually and mathematically. For example, the evolution function of the change detection models has no free parameters whereas the evolution function of free-energy models has 3 free parameters (see Table 2). Critically, because model fit increases monotonically with model complexity, the relative plausibility of these models cannot simply be established based on how well they fit the data. Generally, the true desideratum of model comparison, the generalizability of a model, cannot be determined from fit measures alone; instead, model comparison needs to assess the trade-off between model fit and model complexity [65], [66]. From a Bayesian perspective, this is provided by the (log) model evidence (i.e., the log probability of the data given a model) which corresponds to the negative surprise about the data and represents a principled measure of the balance between model fit and model complexity. Here, we used a Bayesian model selection (BMS) procedure at the group level that treats models as random effects in the population and can successfully deal with population heterogeneity and outliers [35]. As input, this procedure requires the log-evidence of each model considered, for each subject separately. In the following, we describe how these log-evidences were obtained, detailing the likelihood function and priors that underlie the computation of the log-evidence for individual models and subjects.

As EEG signals result from a linear superposition of local electrophysiological responses, one can use a general linear model to map the predictions of local field potentials (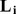 in Table 2) to measured trial-wise MMN amplitudes. In each subject and for each model considered, we modelled the data matrix of trial-wise MMN amplitudes across all trials 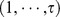 and across all selected electrodes 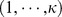 as follows:

Let 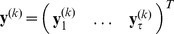 denote the vector of MMN amplitudes at a selected electrode 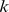. We regard each 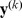 as noisy observations of an electrode-specific linear mixture of evoked neuronal responses that reflect the trial-by-trial evolution of internal states. For each response model 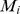 described above, we therefore apply the following multivariate Bayesian linear regression model with conjugate priors to each subject's data:
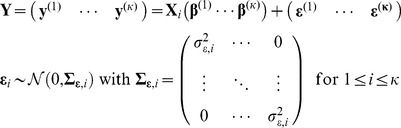
(13)Here, 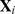 denotes the design matrix that was created by replacing the non-constant columns of 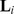 (cf. Eq. 3) by their z-transforms, 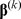 are the regression coefficients for the k^th^ electrode, and 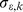 is the standard deviation of measurement errors at the k^th^ electrode. When inverting this model, we used uninformative Gaussian priors on the regression coefficients and uninformative Gamma priors on the error precisions; for details see Section 5 in Text S1.

Note that we are not interested in the regression coefficients but in each model's log-evidence 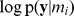. Given the likelihood function and priors described above, the log-model evidences were computed by Monte-Carlo integration (see Section 5 in Text S1 for details). Based on the log model-evidences, we estimated the posterior probability of each model by a Bayesian random effects analysis at the group level [35] with a uniform prior on models. For comparing the model families described in Figure 2 Bayesian inference on partitions of model-space [36] was performed to compute the posterior probability 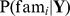 of each model family, where 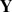 denotes the data across all pre-defined electrodes and subjects. This approach can easily deal with families of different size (i.e., different numbers of models per family). In brief, unbiased family-level inference requires uniform (flat) priors over families, and this was achieved by setting each model's “prior count“ (i.e. the parameters of the Dirichlet prior on model probabilities) to 1 over the size of the respective model family; see [36] for details. Inference on model families used Gibbs sampling with two million samples per family. Finally, we computed the exceedance probability [35] for each model and model family, i.e., the probability that this model (family) was more likely to have generated the data than any other model (family).

## Results

### Models and theories of the MMN

In the Models and Methods section, we derived five classes of models describing how the MMN may reflect the computational processes that govern learning and perception during the roving oddball experiment. Three of the five model classes were derived from the free-energy principle and correspond to formal representations of three contemporary theories of the MMN; i.e., predictive coding, novelty detection, and model adjustment. These models explain the MMN as arising from prediction error signals, surprise or adjustments to model parameters, respectively. Furthermore, we formalized two traditional theories of the MMN: the change detection and adaptation theory. The resulting model space comprised 13 models in five families (see Figure 2). In all models, we have connected the (hidden) processes of perception and learning to measured EEG responses via different response models and a linear electromagnetic forward model. In this section, we assess the relative plausibility of these models and model families using posterior model probabilities and exceedance probabilities computed by Bayesian model selection (BMS) as detailed above. The resulting posterior distributions will be presented as figures, and the main text will report inferences based on those distributions in terms of exceedance probabilities.

**Figure 4:**
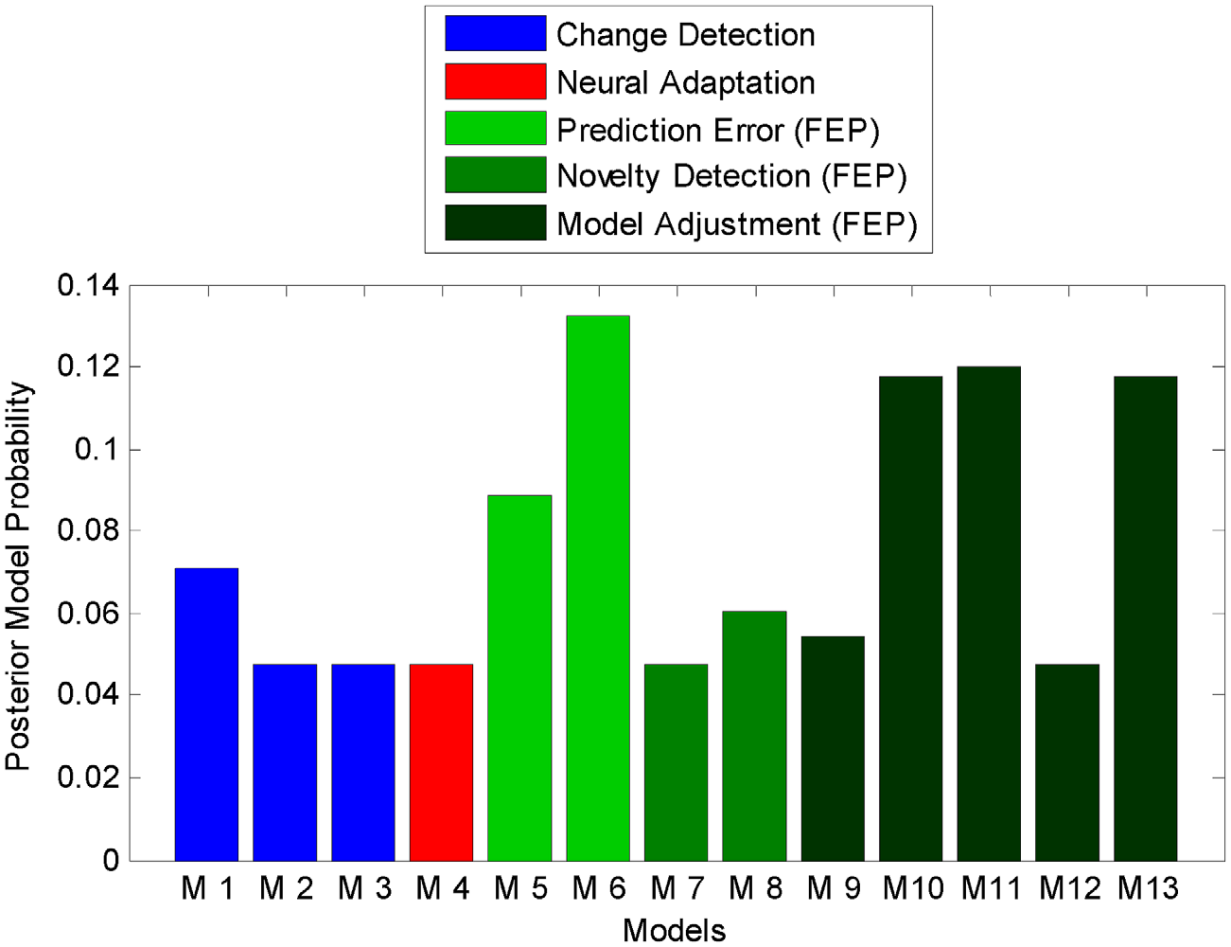
Posterior probabilities of the 13 MMN models. The 13 MMN models were compared by their posterior probability given the trial-wise MMN amplitudes of all eight subjects. These posterior probabilities were computed by random effects Bayesian model selection at the group level. The bars are coloured according to the theory instantiated by each model. The model explaining trial-wise MMN amplitudes by precision weighted prediction errors on the unobservable tone category (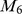) had the highest posterior probability (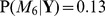). It is closely followed by three almost equally probable “model adjustment” models (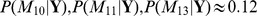), and the model explaining trial-wise MMN amplitudes by prediction errors on the observed log-frequency (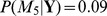).

Figure 4 shows the results of BMS in terms of the posterior probabilities of all models considered. First, note that our “null” model (*M_1_*, the first change detection model), the only model predicting the absence of trial-by-trial changes in MMN amplitudes, is not the best model. Contrary to the predictions of this model, the MMN amplitude appears to vary systematically over deviant trials. This suggests that the MMN is not simply a categorical response to regularity violation but context dependent, as predicted by trial-by-trial statistical learning. Notably, the best five models were all derived within the free-energy framework. Model *M_6_*, which explains trial-wise changes in MMN amplitude as a manifestation of precision weighted prediction errors (on the hidden tone category), was best supported by our data (exceedance probability 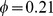). It was followed by three “model adjustment” models (*M_10_*, *M_11_*, *M_13_*), each with exceedance probability 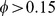. These models explain fluctuations in MMN amplitude as arising from a trial-wise adjustment of the parameters encoding posterior beliefs about the expected number of tone repetitions and the conditional transition probabilities. When examining the fit of the best model, we found that it accounted for 2.3% of the total variance of single-trial MMN amplitudes (across all subjects). The amount of variance explained was significant in each and every subject (p<0.01 in 6 subjects; p<0.02 in two subjects). To put this into perspective, this model-based explanation accounted for about 6.5 times as much variance as could be explained by a more conventional analysis, i.e., a linear regression model considering recent stimulus history (number of standards preceding the deviant).

**Figure 5:**
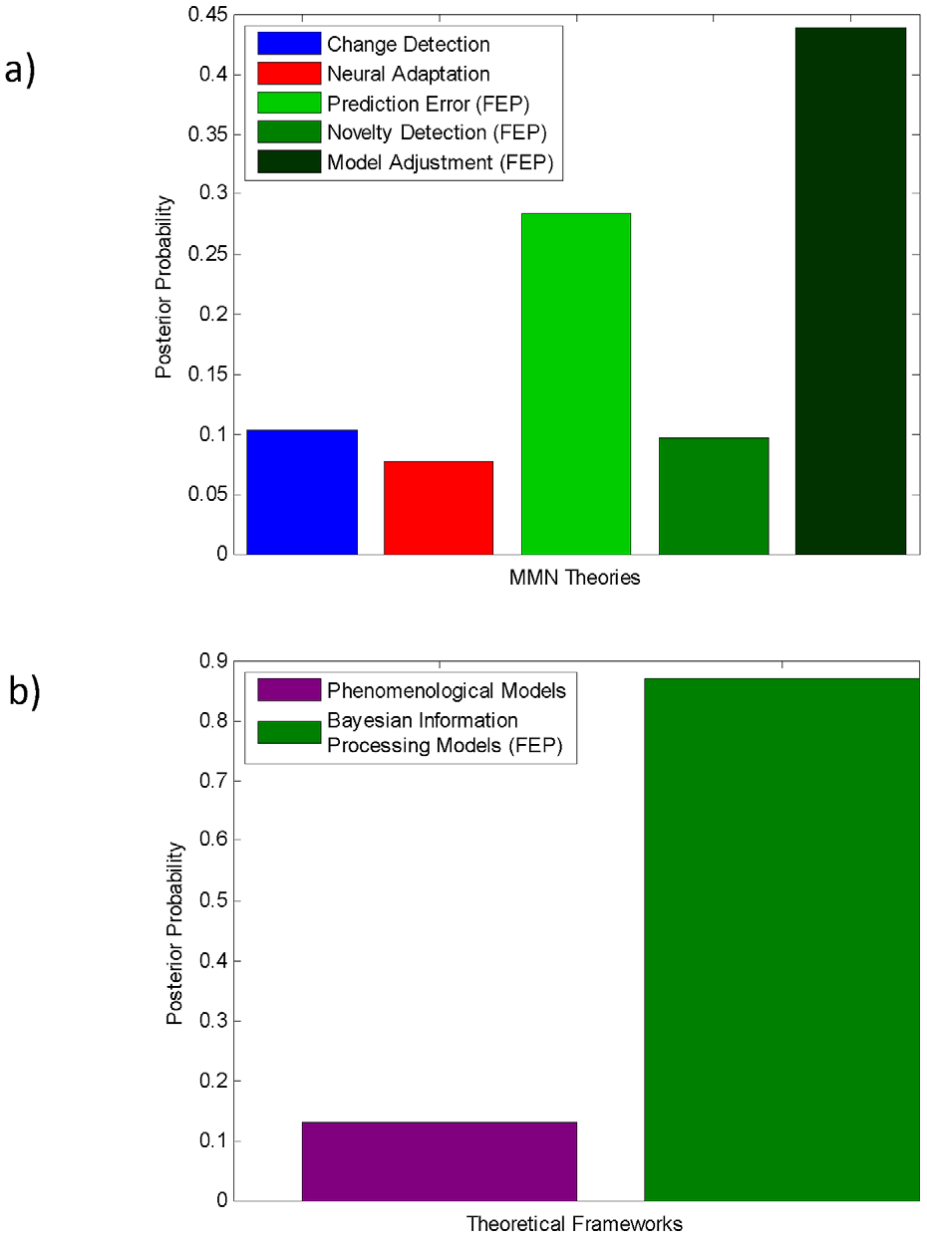
Bayesian model comparison of the five MMN theories (a) and the two frameworks (b). The bar plot in the upper panel (a) summarizes the comparison of the five model families in terms of their posterior probabilities. Each bar indicates the posterior probability of a particular MMN theory (i.e. 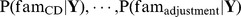). The most plausible explanations of our trial-wise MMN data were provided by the model adjustment hypothesis (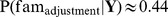) and the prediction error hypothesis (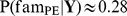). The lower panel (b) shows the results of comparing phenomenological (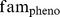) vs. free-energy based models (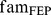); see Figure 2. It shows that our free-energy based models provide considerably more convincing explanations of our MMN data than traditional change detection or adaptation models (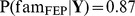).

While the exceedance probability of the best model M_6_ was about five times as large as the exceedance probability of our “null” model M_1_, this was too small to yield an acceptably low probability of model selection error [67]. As the bar plot shows, the probability mass is concentrated on two model families (prediction error and model adjustment) but distributed over several models. Thus, BMS at the level of model families was more appropriate than comparing individual models. From a statistical perspective, this trades a reduced resolution of the hypothesis (model) space for increased statistical power. In other words, we move from asking which specific model is best to asking which of the five general MMN theories best explains the data, irrespective of their precise implementations (cf. Figure 2). This comparison of the five model families is summarized in Figure 5a. The most plausible MMN theory was the model adjustment theory (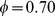), followed by the prediction error theory (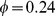).

Finally, we used BMS to examine whether the free-energy principle based models provide, in general, better explanations of the variability of single-trial MMN amplitudes than phenomenological models. This means we are now comparing only two families (Figure 2): the family of free-energy based models (predictive coding, novelty detection and model adjustment; 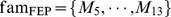) and the family of more traditional phenomenological models (change detection and adaptation, 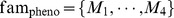). Family-level BMS indicated that models based on the free-energy principle were considerably more convincing than phenomenological models; 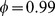 (see Figure 5b).

### Level of representation

Finally, we asked which level of the processing hierarchy contributes most to the fluctuations in trial-wise MMN amplitudes. In other words, we examined whether response variations arise from lower auditory areas representing physical sound properties like frequency, or from higher areas that represent abstract temporal structure. For this purpose we re-partitioned the 13 models into two families according to whether they explain MMN generation in relation to a low-level auditory feature (sound frequency) or a high-level auditory feature (temporal structure). For the models based on the free-energy principle models the two levels of representation map onto the two levels of the mental model: sensory inputs and hidden sequence of tone categories (Figure 3). We assigned the free-energy based models that relate the MMN elicited by changes in sound frequency to the representation of sound frequencies to the first model family and those that relate it to the represented sequence of tone categories to the second. Furthermore, both the adaptation model and the change detection theory are formulated explicitly with regard to stimulus frequencies and are therefore assigned to the first model family. Overall, this resulted in the following two model families: 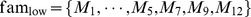 and 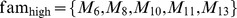. Comparing these two model families yielded an exceedance probability of 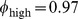 for 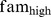, suggesting that the auditory MMN is more closely related to a representation of high-level auditory features, such as temporal structure, than to a representation of low-level features, such as sound frequency.

### Single-trial MMN amplitudes are history-dependent

**Figure 6:**
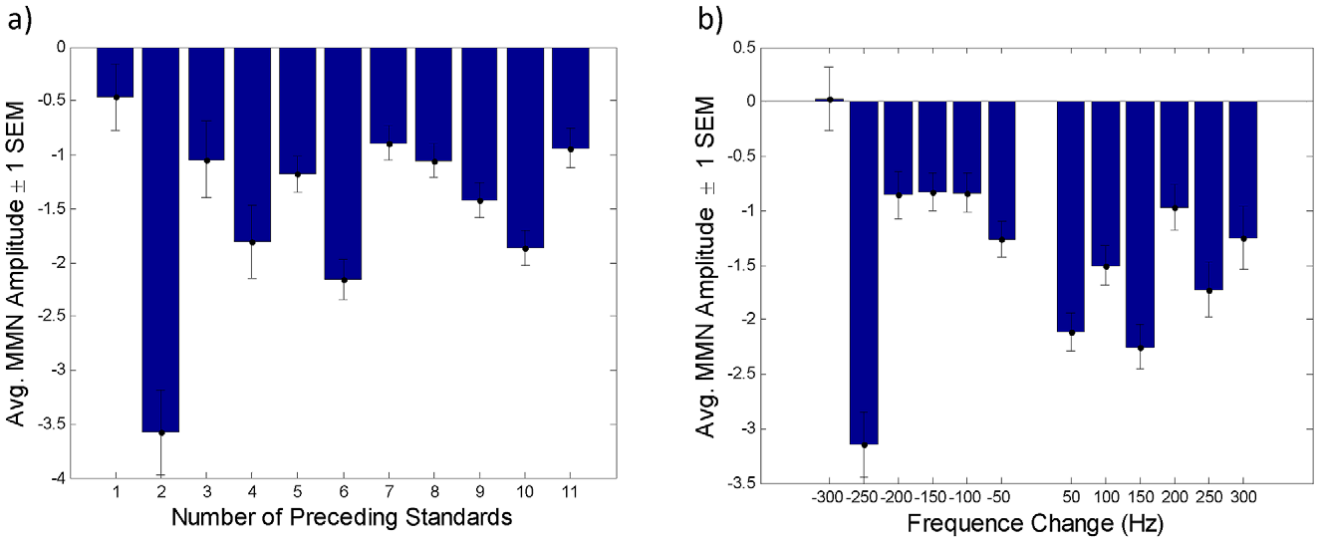
Trends in MMN amplitudes. Figure 6a shows the average MMN amplitude as a function of the number of standards preceding the deviant. Figure 6b shows the average MMN amplitude as a function of the frequency of the deviant minus the frequency of the preceding standard (frequency change).

The models reported above were designed to predict the evolution of single-trial MMN amplitudes throughout the experiment. This was done to capture putative history-dependent effects. The models which did take into account such effects (i.e., free energy based models) were found to have higher evidence than models which did not (e.g., the various change detection models). One may ask, however, as did one of our reviewers, whether our single-trial approach was really necessary or whether it would have been sufficient to analyse the *average* MMN amplitude as a function of the number of preceding standards and the change in frequency. Here we provide a conventional analysis of variance to demonstrate that our data did contain history-dependent effects that would have been removed by conventional averaging. By history-dependent effects we mean that the MMN amplitude evoked by a deviant following a given number of standards and a given frequency change will differ depending on the tones that preceded the current sequence of standards. The mere number of such tones is a minimal definition that ignores the effects of their statistical structure, some of which are captured by our models. However, it allows for a conservative test of history-dependence, i.e., whether a 3-way analysis of variance (ANOVA) of trial-wise MMN amplitudes reveals interactions among three factors: (i) number of preceding standards, (ii) frequency difference, and (ii) time, i.e., the number of preceding trial sequences. We found significant main effects for the number of preceding standards and for frequency difference (Figure 6). More importantly, however, we found highly significant interaction effects, indicating that the effect of the number of preceding standards on MMN amplitude did not only depend on the frequency difference between standard and deviant (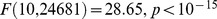) but also on the number of previous tone sequences (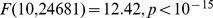). This demonstrates that the trial-wise MMN amplitudes we recorded do indeed show history-dependent effects that would be removed by conventional averaging procedures.

## Discussion

In this paper we presented a framework for modelling single-trial responses, applied it to formalize five major theories of the MMN (see Figure 2), and tested them quantitatively against trial-wise MMN amplitudes measured with EEG from eight healthy volunteers. Our main finding was that models linking the MMN to computations approximating Bayes-optimal sensory learning and perception (see Figure 3) provide better predictions of single-trial MMN amplitudes than two classical theories (see Figure 5). Furthermore, this paper offered a unifying perspective on three current theories of the MMN: prediction errors, model adjustment, and novelty can all be seen as manifestations of approximate Bayesian learning of sensory regularities by free-energy minimization.

### Single-trial MMN amplitudes are informative about statistical learning

Our analyses suggested that stimulus history (i.e., previous tone sequences) affects the MMN in intricate ways. This was not only demonstrated by a simple ANOVA of single-trial MMN amplitudes, but, more importantly, by our systematic model comparisons which favoured free-energy based Bayesian information processing models that capture history-dependent effects. In particular, these models explain the dependence of the MMN on interactions between previous tone sequences and the current tone sequence in terms of trial-by-trial learning of statistical structure. Trial-by-trial statistical learning implies that the probabilistic expectation evoked by a given tone sequence is different for every presentation, and that each difference reflects what has been learned since the previous presentation. While traditional MMN studies have ignored trial-specific effects by averaging responses across deviant events, several studies have addressed sequential changes in the MMN across trials [19], [23], [25]–[27], [51]–[54], [68]–[70]. However, only [23] and [68] have completely avoided averaging procedures altogether. The results of this study and [23] question the frequent assumption that the MMN amplitude is constant throughout an experimental condition (i.e., for given tones and following a given number of standards). Instead, our results suggest that trial-by-trial changes in MMN amplitude are highly history-dependent and represent an informative index of statistical learning as the recording session proceeds. It is pleasing that [23] reached a similar conclusion, even though they studied mismatch potentials in a different modality (i.e., somatosensory) and with simpler models, but with source-reconstruction and a high temporal resolution. Thus, while averaging is a useful tool to increase the signal-to-noise ratio, single-trial data carry unique information about the processes of learning and perception that underlie the MMN.

### MMN amplitude as a function of the number of preceding standards

A number of previous studies reported that the MMN amplitude elicited by a change in sound frequency increases monotonically with the number of preceding standards [27], [51]–[54], [69], [70]. By contrast, we found a non-monotonic effect of the number of preceding standards on deviant response amplitude (see Figure 6a). The reason for this discrepancy may be that previous studies did not disentangle the contributions of the standard ERP and the deviant ERP (cf. [71]). In contrast, in this study, we operationalized the MMN with respect to a fixed standard ERP (see *Models and Methods*), so that changes in MMN amplitude reflected changes in the neural response to the deviant only. In summary, our results do not contradict previous findings on the relationship between the number of preceding standards and the MMN amplitude [27], [51]–[54], [69], [70] but complement them. Furthermore, our models based on the free-energy principle can explain why Haenschel et al. [27] observed a monotonic decay of the standard response with the number of standard repetitions, and they predict how stimulus history determines the effect of preceding standards on deviant response amplitude.

### Implications for theories of the MMN

Our modelling results do not lend support to the adaptation hypothesis of the MMN [18] or the change detection interpretation of the memory trace hypothesis [72]. Instead, our results support explanations postulating that the brain maintains and constantly updates an internal model of its environment. For example, the *model adjustment* hypothesis [19] posits that auditory cortex maintains a model of the acoustic environment, and that stimulus-induced updates of this model are indexed by the MMN [20]. While the original proposal was of a conceptual nature, our present work formalizes this hypothesis by specifying *how* trial-wise changes in MMN reflect an approximation to Bayesian updating of a probabilistic mental model. The resulting models are consistent with the conclusion drawn by [23] that (somatosensory) mismatch potentials reflect perceptual learning. However, our analysis was more fine-grained in that it distinguished between three computational mechanisms that might underlie the perceptual learning that [23] indexed in terms of Bayesian surprise. Concretely, we distinguished between prediction error signalling, novelty detection, and model adjustment. Our results supported model adjustment and, to a lesser extent, prediction error signalling, but not novelty detection, even though it computes an approximation to (Shannon) surprise. We also distinguished between perceptual learning at the level of physical stimulus properties (sound frequency) and learning of abstract temporal structure and found strong evidence for the latter. In neurobiological terms, model adjustment might correspond to synaptic plasticity at top-down projections targeting pyramidal neurons in layers 2 and 3 (“prediction error units”) via NMDA receptors [3] (see Section 6 in Text S1). This would be consistent with the observation that pharmacological blockage of NMDA receptors diminishes the MMN [73]–[75].

Predictive coding formulations of free-energy minimization assign prediction errors a critical role in the update of posterior beliefs. When comparing all models individually, the best model was indeed one that explained trial-wise fluctuations in MMN amplitude as a function of precision weighted prediction errors (model M_6_; Figure 4). However, its superiority over other models was marginal, and model comparison at the family-level (Figure 5a) did not support the hypothesis (proposed in [3]) that the MMN solely reflects precision weighted prediction errors. This suggests that while prediction error signalling may be essential for the free-energy minimization process underlying the MMN, it is probably not the sole determinant of trial-wise MMN amplitudes. Alternatively, our failure to find stronger evidence for the hypothesis that (precision weighted) prediction errors alone determine trial-wise MMN amplitudes may be due to some of our simplifying assumptions, as discussed in the next section.

### Limitations

Overall, one should bear in mind that our inferences are primarily about rather abstract models or classes of models. Our free-energy based models, in particular, consider the outcomes of neuronal computations rather than their process. This is a necessary constraint on models of discrete trial-by-trial variations in responses; as opposed to continuous time models that would consider the precise time-course of neural responses over peristimulus time. This means that we have to assume that there is some aspect of neuronal activity or excitability that encodes the posterior beliefs associated with each oddball trial. However, the relationship between biophysical quantities like synaptic activity or gain, on the one hand, and posterior beliefs, predictions, and surprisal, on the other hand, are not specified explicitly in this sort of model. This means that it is difficult to make any strong statements about the neurobiology that implements any Bayesian inference.

Furthermore, our models make several simplifying assumptions that may turn out to be false. First, there is still no conclusive evidence about how prediction errors are represented at the level of single neurons. Second, the assumption of a linear relationship between the encoded quantity and the MMN amplitude is simplistic and ignores potential nonlinearities. Third, all of our models represent the MMN by a single number (i.e., its peak amplitude), rather than by its waveform, thereby ignoring its temporal dynamics and spatial topography. Fourth, each of our models relates trial-wise MMN amplitudes to a single computational variable, whereas it is known that the MMN scalp potential is a mixture of signals from several brain areas with (presumably) different functional characteristics [29]–[31], [76]. Finally, while our results indicated that our neuronal adaptation model M_4_ is insufficient to explain single-trial variations in MMN, we have not tested the fresh-afferent theory [13] that is based on stimulus specific adaptation. In future work, it would be useful to formulate this theory as models of stimulus specific adaptation [12], [13], [42] under the present framework and compare it to the computational models presented in this paper.

### Relation to the Bayesian-brain hypothesis

Our models based on the free-energy principle link the MMN to the neuronal encoding of posterior beliefs that is postulated by the *Bayesian brain hypothesis*. According to this hypothesis, the brain represents probabilistic beliefs, and updates them in an (approximately) Bayesian fashion. Previous work along these lines has assumed that the support of probability distributions is partitioned into small bins and that each bin's probability mass is represented by the firing rate of dedicated neurons [77], [78], or that probability densities are approximated by a linear combination of basis functions [79]. In contrast to these high-dimensional representations, we have implicitly assumed a much simpler, low dimensional fixed-form approximation to the posterior density. Our predictors of electrophysiological responses are simple functions of posterior expectations on log-frequency, tone category and transition probabilities. These posterior expectations might be encoded by the average activities of neuronal populations, and the precision parameters that determine the relative weight assigned to prior beliefs and sensory evidence could be encoded by the strength of the recurrent connections of prediction error units [80] (see also Section 6 in Text S1). This representation is not motivated by sparseness, but by computational efficiency: It replaces the problem of computing the (potentially very high-dimensional) posterior probability density by optimizing the free-energy with respect to a small set of sufficient statistics. This variational Bayesian optimization rests on free-energy minimization [37] and proposes the minimization of prediction error as an explanation for stimulus-evoked transient neuronal responses such as the MMN [3], [63], [81]. The work presented in this paper is a step towards linking models of probabilistic neural coding and inference to neuronal signals that can be measured non-invasively in humans.

### Potential future directions

Our present results were based on a single “roving oddball” EEG experiment that was originally designed for comparing dynamic causal models of interactions among cortical areas during the MMN [25]. In the future, it would be interesting to apply the approach presented here to other types of MMN paradigms. Additionally, one could use our models in conjunction with recent advances in design optimization that maximize the sensitivity of Bayesian model selection [67] to create an experiment that is optimal for discerning between the models selected by our analysis. In addition, our modelling and model comparison framework could be applied to source-reconstructed mismatch potentials to characterize functional differences between the brain areas jointly generating MMN scalp potentials.

Furthermore, the link between single-trial mismatch potentials, on the one hand, and statistical learning and perceptual inference, on the other hand, could be exploited to measure the temporal dynamics of how the brain learns the probabilistic structure of complex environments. This is an attractive prospect, given that the MMN is elicited not only in simple oddball experiments, but also in more complex experiments involving speech, language, music, and abstract features, as well as various other sensory modalities [14], [71], [82], [83]. Our modelling framework could also be used to probe disturbances of perceptual inference and learning in psychiatric conditions, such as schizophrenia [84]–[86]. In addition, future studies might use the meta-Bayesian approach [34] for inferring, from single-trial MMN amplitudes, subjects' prior beliefs about hidden temporal structure, which constitute the inductive biases [87] that endow the brain with its remarkable ability to discover complex sequential regularities.

## Supporting Information

Text S1 **Mathematical details of our** **models and methods** **.** Sections 1—4 provide additional information about the models based on the free-energy principle. Concretely, these sections specify how we modelled the brain's internal model of tone sequences, learning and perception, individual differences, and the manifestation of neurocomputational variables in scalp potentials. Section 5 explains how we approximated each model's log-evidence. Section 6 sketches how the computations postulated by the free-energy models could be implemented in the brain.
(PDF)
