# Supplementary material for: Modelling Trial-by-Trial Changes in the Mismatch Negativity
Source: PLoS Comput Biol. 2013 Feb 21;9(2):e1002911. doi: 10.1371/journal.pcbi.1002911 (PMC3578779; doi:10.1371/journal.pcbi.1002911)
Supplement: Text S1 — Mathematical details of our models and methods . Sections 1—4 provide additional information about the models based on the free-energy principle. Concretely, these sections specify how we modelled the brain's internal model of tone sequences, learning and perception, individual differences, and the manifestation of neurocomputational variables in scalp potentials. Section 5 explains how we approximated each model's log-evidence. Section 6 sketches how the computations postulated by the free-energy models could be implemented in the brain. (PDF) [file pcbi.1002911.s001.pdf]

Text S1

1. The Bayesian observer's probabilistic mental model

The information processing models presented in the main text are based on a Bayesian observer that perceives and learns about tone sequences according to approximate Bayesian inference in a probabilistic mental model m. This section specifies this probabilistic model m in detail.[1]

The main text defined the probabilistic mental model m by the following set of assumptions about the observations $\mathbf{u}$, hidden states $\mathbf{z}$, and parameters $\vartheta = \{\omega, \eta, a\}$:

$$\begin{gathered}
\mathrm{p}(\mathbf{u}, \mathbf{z}, \vartheta \mid m) = \mathrm{P}(z_0) p(u_0 \mid z_0, \vartheta) \left(\prod_{t=1}^{T} \mathrm{P}(z_t \mid \mathbf{z}_{1:t-1}, \vartheta) \mathrm{p}(u_t \mid z_t, \vartheta) \right) \mathrm{p}(\vartheta; \boldsymbol{\theta}) \\
\mathrm{P}(z_0) = \mathrm{Uniform}(\{1, \cdots, c\}) \\
\mathrm{P}(z_{t+1} = j \mid z_t = k, \mathbf{z}_{1:t-1}, \eta, a) = \mathrm{T}_{k,j}(\mathbf{z}_{1:t-1}, \eta, a) \\
\mathrm{p}(u_t \mid z_t; \vartheta) = \mathcal{N}(\omega_{z_t}, \sigma^2) \\
\vartheta = \{\omega, \eta, a\}, \mathrm{p}_{\boldsymbol{\theta}}(\vartheta)
\end{gathered} \tag{1}$$

The observations $\mathbf{u}$ are log-frequencies, the hidden states $\mathbf{z}$ are tone categories, T is a matrix-valued function returning the transition probabilities between hidden states. The parameters $\vartheta = \{\omega, \eta, a\}$ denote the mean frequencies of the c tone categories ($\omega_1, \cdots, \omega_c$), the expected number of tone repetitions (η), and the conditional transition probabilities given the hidden state changes (a), respectively. The hyperparameters $\boldsymbol{\theta} = \{\sigma, n_0, \eta_0\}$ specify the observer's perceptual uncertainty σ^2, the prior expectation on the number of tone repetitions (η_0), and the strength of the prior beliefs (n_0). Please see Figure 3 of the main text for an illustration of the probabilistic mental model and its role within our free-energy models and Table 2 of the main text for the definitions of the variables used to describe the evolution function and the response function.

After this brief summary of the definition given in the main text, we will first describe the structure of the transition matrix $\mathrm{T}(\mathbf{z}_{1:t-1}, \eta, a)$ and its dependence on the parameters $\eta, a \in \vartheta$ and the history of hidden states $\mathbf{z}_{1:t-1}$. Second, we will describe the priors $\mathrm{p}_{\boldsymbol{\theta}}(\vartheta)$ on the parameters ϑ of the mental model m.

---

[1] In terms of notation, the colon in the subscript of a variable, e.g. $\mathbf{u}_{a:b}$, is used to refer to the sequence of its values from trial number a to trial number b. Furthermore, a colon by itself denotes the set of all values, e.g., $\mathbf{x}_{:,t}$ represents all elements of $\mathbf{x}$ on trial t.

Structure of the transition matrix

The transition matrix $\mathrm{T}(\mathrm{z}_{1:t};\eta,a)$ of the probabilistic mental model m is structured into the probability

that the category will stay the same (s_r) and the conditional probabilities of transitions to other categories given that the category changes ($\mathbf{a}$). Importantly, the probability for a category to stay depends on the history of previous states ($\mathbf{z}_{1:t-1}$) via the number of times the current state has been repeated since the last change in category ($R=r$). Specifically, the probability of a category to stay the same depends only on r but not on the category itself. By contrast, the conditional probability that the next state will be k if the current state is i ($a_{i,k}$) does not depend on this history, but only on the two categories (i,k).

$$\mathrm{T}_{i,k}(\mathrm{z}_{1:t};\eta,a)=\begin{cases} s_r \text{ if } i=j \\ a_{i,k}\cdot(1-s_r) \text{ if } i\neq j \end{cases}, \quad r: z_{t-r-1}\neq z_t \wedge \forall 0\leq l\leq r: z_{t-l}=z_t \tag{2}$$

We model the subject's belief about how probable it is that a category will be repeated r times by a Poisson distribution ($\mathrm{P}(R=r)=\mathrm{Poisson}(r;\eta)$), and the subjective probability that a category will stay the same follows from this belief:

$$R\sim\mathrm{Poisson}(\eta),\ s_r=\left(1-\mathrm{P}(R\leq r)\right)/\prod_{i=1}^{r-1}s_i \tag{3}$$

Prior Beliefs about the Parameters of the Mental Model

As the previous section shows, the transition matrix can be parameterized by how often the current tone has been repeated, the expected number of tone repetitions (η) and the conditional transition probabilities ($\mathbf{a}$). Choosing conjugate priors on these parameters of the subjects' mental model, the prior on the expected number of repetitions (η) is a Gamma distribution (the likelihood is Poisson, see above). Its strength corresponds to n_0 virtual observations and its mean is η_0.

$$P(\eta)=\mathrm{Gamma}(\gamma_0,\delta_0),\ \gamma_0=\mathrm{n}_0\cdot\eta_0,\delta_0=n_0 \tag{4}$$

The priors on the conditional transition probabilities ($a_{i,k}$) are Dirichlet distributions. Their parameters have been chosen to reflect an equal number of virtual observations of transitions from each of the c tone categories (n_0/c) to each of the $c-1$ other tones ($(n_0/c)/(c-1)$). The existence of the mode was ensured by adding one. In effect, the prior modes of the conditional transitions probabilities are $1/(c-1)$ for each transition.

$$\mathrm{P}(\mathbf{a}_{i,:})=\mathrm{Dirichlet}(:,\boldsymbol{\alpha}_{i,:}),\forall i\neq j:\alpha_{i,j}=(n_0/c)/(c-1)+1 \tag{5}$$

The prior belief about how the hidden states generate observations is captured by a Gaussian prior on the categories' mean frequencies (ω).

$$\begin{aligned} &\mathrm{P}(\omega) = \prod\nolimits_{i=1}^{c} \mathcal{N}\left(\mu_i, \sigma^2 \big/ \eta_0 \cdot n_0 / c \right), \; c = 10 \\ &\mu = \log\left(493.9, 523.3, 554.4, 587.3, 622.3, 659.3, 698.5, 740.0, 784.0, 830.6\right)\mathrm{Hz} \end{aligned} \tag{6}$$

The mental model m detailed in this section determines how the observer's perceives and learns from sensory input. The following section details how learning and perception were modeled in the Bayesian observer's evolution function (f_{FEP}, see Figure 3 of the main text).

2. Perception and Learning by Free-Energy Minimization

We minimized free-energy by the following two-step procedure that is similar to the Expectation-Maximization (EM) algorithm[1]. In short, we computed two variational updates for each observation. The first update corresponds to perception (E-step), and the second update corresponds to learning (M-step). In our simulations this procedure was sufficient for veridical perception, but more complex stimuli and models would require several iterations of the two steps.

Both updates can be derived by minimizing the following explicit form of the free-energy (see Eq. 11 in the main text):

$$\begin{aligned} &\mathcal{F} = -\ln \mathrm{p}(\mathbf{u}, \mathbf{x}_{\mathbf{z},:}, \mathbf{x}_{\vartheta,:} \mid m) \\ &= -\ln(1/c) - \ln N(u_1 \mid \mathbf{x}_{\omega,2}(x_{z,2}), \sigma^2) - \ln p_\theta(\mathbf{x}_{\vartheta,\mathbf{n}_t+1}) + \cdots \\ &\left(\sum\nolimits_{t=2}^{n_t} -\ln P(z_t = x_{z,t+1} \mid z_{t-1} = x_{z,t}, \mathbf{z}_{\mathbf{1:t\text{-}2}} = \mathbf{x}_{\mathbf{z,2:t\text{-}1}}, \eta = x_{\eta,t}, a = \mathbf{x}_{\mathbf{a,t}}) - \ln N(u_t \mid \mathbf{x}_{\omega,\mathbf{t+1}}(x_{z,t+1}), \sigma^2) \right) \end{aligned} \tag{7}$$

Variational Update 1: Posterior Expectations of Hidden States

In the first step, the posterior expectation of the hidden state z_t (i.e. $x_{z,t+1}$) is inferred by minimizing free-energy under the previous trial's approximate posterior belief about the parameters ($\vartheta = x_{\vartheta,t}$):

$$x_{z,t+1} = \arg\min_{\mathbf{z}} \left\langle -\ln p(u_t, \mathbf{z}, \vartheta \mid \mathbf{x}_{\mathbf{z,1:t}}, m) \right\rangle_{q_{\vartheta,t}} \tag{8}$$

Because the hidden state can only take finitely many values, this optimization problem can be solved by computing the free-energies associated with each of the categories and choosing the one for which the free-energy is lowest.

$$\begin{aligned} &x_{z,t+1} = \arg\min_c \mathcal{F}_z(c,t) \\ &\mathcal{F}_z(c,t) = \begin{cases} -\ln(1/C) - \ln N(u_t; x_{\omega,t}(c), \sigma^2) & \text{if } t=1 \\ -\ln(s_r) - \ln N(u_t; \mathbf{x}_{\omega,\mathbf{t}}(c), \sigma^2) & \text{if } t>1,\ c = x_{z,t} \\ -\ln(1-s_r) - \ln(\mathbf{x}_{\mathbf{a,t}}(c, x_{z,t})) - \ln N(u_t; \mathbf{x}_{\omega,\mathbf{t}}(c), \sigma^2) & \text{if } t>1,\ c \neq x_{z,t} \end{cases} \end{aligned} \tag{9}$$

Variational Update 2: Posterior Expectations of the Parameters

The posterior expectations on the parameters were computed by optimizing the free-energy under the posterior expectations on the hidden states.

$$x_{\vartheta,t+1} = \arg\min_{\vartheta} \left\langle -\ln \mathrm{p}(u_t, x_{z,t+1}, \vartheta \mid x_{z,1:t}, m) \right\rangle_{q_{z,t+1}} \tag{10}$$

The structure of the mental model m implies that the three types of parameters are conditionally independent given the hidden states. Therefore their posterior expectations can be found independently of each other; this is described in the following three paragraphs. Since the prior probability densities on these parameters are conjugate to the corresponding likelihood functions, we can exploit that the free-energy minimizers are equal to maximum-a-posteriori estimates. Furthermore, the optimization can ignore additive constants that do not depend on the parameter in question.

1. *Posterior expectation of the category's mean frequency*

Minimizing free-energy (Eq. (7)) with respect to the mean frequency of the inferred category is equivalent to minimizing the sum of the negative logarithm of its prior density and the negative logarithm of the density of the observations assigned to its category. These two densities correspond to a Gaussian prior and a Gaussian likelihood respectively. Therefore, the Gaussian posterior distribution can be computed analytically and its mode is the free-energy minimizer w.r.t. the category's mean frequency.

$$\begin{aligned}
&\mathbf{x}_{\boldsymbol{\omega},\mathbf{t}+1}(\tilde{c}) = \arg\min_{\boldsymbol{\omega}(\tilde{c})} \mathcal{F}_{\boldsymbol{\omega}}(\boldsymbol{\omega}, t) \\
&\mathcal{F}_{\boldsymbol{\omega}}(\boldsymbol{\omega}(\tilde{c}), t) = -\ln N(\boldsymbol{\omega}(\tilde{c}); \mu_{\tilde{c}}, \sigma_{\omega}^2) - \sum_{i \le t} \mathbf{1}_{x_{z,i+1}=\tilde{c}} \cdot \ln N(u_i; \boldsymbol{\omega}(\tilde{c}), \sigma^2) \\
&\mathbf{x}_{\boldsymbol{\omega},\mathbf{t}}(\tilde{c}) = \arg\max_{\boldsymbol{\omega}(\tilde{c})} N\left(\boldsymbol{\omega}(\tilde{c}); \boldsymbol{\mu}_{\mathbf{post}}(\tilde{c}), (\sigma_{\omega}^{-2} + n_{\tilde{c}} \cdot \sigma^{-2})^{-1}\right) = \boldsymbol{\mu}_{\mathbf{post}}(\tilde{c}) \\
&\boldsymbol{\mu}_{\mathbf{post}}(\tilde{c}) = \frac{\sigma_{\omega}^{-2} \cdot \mu_{\tilde{c}} + \sigma^{-2} \cdot \sum_{i \le t : x_{z,i+1}=\tilde{c}} u_i}{\sigma_{\omega}^{-2} + n_{\tilde{c}} \cdot \sigma^{-2}}, n_{\tilde{c}} = \sum_{i \le t} \mathbf{1}_{x_{z,i+1}=\tilde{c}}
\end{aligned} \tag{11}$$

2. *Posterior expectation of the expected sequence length*

We assume that the belief about sequence lengths is updated only when a transition occurs. Minimizing free-energy in Eq. (7) with respect to the expected sequence length is equivalent to minimizing the sum of the negative logarithm of its prior density and the negative logarithm of the density of the observed sequence length. These two densities correspond to a Gamma prior and a Poisson likelihood, respectively. Therefore, the posterior is a Gamma distribution and can be computed analytically. Its mode is the free-energy minimizer w.r.t. the expected sequence length:

$$
\begin{aligned}
&x_{\eta,t+1} = \arg\min_e \mathcal{F}_\eta(e,t) \\
&\mathcal{F}_\eta(e,t) = -\ln \mathrm{Gamma}(e; n_0 \cdot \eta_0, n_0) - \sum_{1<i\le t} \mathbf{1}_{x_{z,i+1} \ne x_{z,i}} \cdot \ln \mathrm{Poisson}(r_i; e) \\
&x_{\eta,t} = \arg\max_e \mathrm{Gamma}\left(\mathrm{e}; \gamma_{\mathrm{post}}, \delta_{\mathrm{post}}\right) = \frac{\gamma_{\mathrm{post}} - 1}{\delta_{\mathrm{post}}} \\
&\gamma_{\mathrm{post}} = n_0 \cdot \eta_0 + \sum_{1<i\le t} \mathbf{1}_{x_{z,i+1} \ne x_{z,i}} \cdot r_i, \delta_{\mathrm{post}} = n_0 + \sum_{1<i\le t} \mathbf{1}_{x_{z,i+1} \ne x_{z,i}}
\end{aligned}
\tag{12}
$$

3. *Posterior expectation of the conditional transition probability*

The belief about the conditional transition probabilities from a given state is updated whenever a transition away from this state has been inferred. Minimizing free-energy in Eq. (7) with respect to the conditional transition probabilities is equivalent to minimizing the sum of the negative logarithm of their prior density and the negative logarithm of the density of the observed transition. These two densities correspond to a Dirichlet prior and a Multinomial likelihood respectively. Therefore, the posterior is again a Dirichlet distribution and can be computed analytically. Its mode is the free-energy minimizer w.r.t. the conditional transition probabilities.

$$
\begin{aligned}
&\mathbf{x}_{\mathbf{a},\mathbf{t}+1}(k,:) = \arg\min_{a_{k,:}} \mathcal{F}_a(\mathbf{a}_{\mathbf{k},:}, t) \\
&F_a(\mathbf{a}_{\mathbf{k},:}, t) = -\ln \mathrm{Dirichlet}(\mathbf{a}_{\mathbf{k},:}; \boldsymbol{\alpha}_{\mathbf{k},:}) - \sum_{1<i\le t} \mathbf{I}_{x_{z,i+1} \ne x_{z,i} = k} \cdot \ln \mathrm{Multinomial}(x_{z,i+1}; \mathbf{a}_{\mathbf{k},:}) \\
&\mathbf{x}_{\mathbf{a},\mathbf{t}}(k,:) = \arg\max_{\mathbf{a}_{\mathbf{k},:}} \mathrm{Dirichlet}(\mathbf{a}_{\mathbf{k},:}; \boldsymbol{\alpha}_{\mathbf{k},:} + \mathbf{n}_{\mathbf{k},:}) = \begin{pmatrix} \dfrac{\alpha_{k,1} + n_{k,1} - 1}{n_{\mathrm{total}} - (c-1)} & \cdots & \dfrac{\alpha_{k,c-1} + n_{k,c-1} - 1}{n_{\mathrm{total}} - (c-1)} \end{pmatrix} \\
&n_{k,l} = \sum_{1<i\le t} \mathbf{I}_{l = x_{z,i+1} \ne x_{z,i} = k}, n_{\mathrm{total}} = \sum\nolimits_{l \ne k} (\alpha_{k,l} + n_{k,l})
\end{aligned}
\tag{13}
$$

The recognition and learning processes derived in this section depend upon three hyperparameters ($\boldsymbol{\theta} = \{\sigma^2, n_0, \eta_0\}$). These hyperparameters are subject-specific and have to be estimated as discussed in the following section.

3. Modeling individual differences

Inter-individual differences in learning and inference are modeled through different settings of the hyper-parameters ($\boldsymbol{\theta}$) of the evolution function ($\mathrm{f}_{\mathrm{FEP}}$) introduced in the main text:

$$
\mathrm{x}_{\mathrm{t+1}} = \mathrm{f}_{\mathrm{FEP}}(\mathrm{x}_{\mathrm{t}}, u_t; \boldsymbol{\theta}) = \arg\min_{\mathrm{x}_{\mathrm{t+1}}} \mathcal{F}(\mathrm{x}_{\mathrm{t+1}}, u_t, \mathrm{x}_{\mathrm{t}}, m) \tag{14}
$$

They do not describe the environment represented by the observer's mental model (m) but fixed properties of the observer that determine how he or she processes information [2]. We do not make strong assumptions about these parameters, because we are not aware of any empirical findings that would justify them. Instead, we estimate each subject's hyperparameters from his or her data. The three hyperparameters ($\boldsymbol{\theta} = \{\eta_0, n_0, \sigma^2\}$) fully determine the prior beliefs about the probabilistic structure of the environment that a subject brings to the experiment. The strength of the subject's prior beliefs is

captured by the fictitious number of tone sequences heard before the experiment (n_0). This hyperparameter determines how fast the observer will learn from experience. This one parameter is sufficient to parameterize the observer's initial belief about the conditional transition probabilities (Eq. (5)) because we assume that all changes are equally probable a priori. The second hyperparameter (η_0) is the a priori expectation of the sequence length. Together with n_0 it is sufficient to parameterize the observer's belief about the expected number of tone repetitions (4). The third hyper-parameter (σ^2) quantifies the observer's perceptual uncertainty ($u_t \sim N(\omega_{Z_t};\sigma^2)$).

The hyperparameters $\boldsymbol{\theta}$ are assumed to vary across subjects. In order to estimate these parameters, one has to postulate a prior on these parameters. The prior we have chosen is uninformative about the scale of the hyperparameters across a wide, but finite, range of possible values. The support of the prior was limited to a finite range, because model selection requires proper priors.

$$
\begin{aligned}
&P(\boldsymbol{\theta} \mid M_i) = P_n(n_0) \cdot P_\eta(\eta_0) \cdot P_\sigma(\sigma^{-2}) : \forall i \in \{5, \cdots, 13\} \\
&P_\sigma(\cdot) = P_n(\cdot) = P_\eta(\cdot) = \text{Uniform}(\log(\cdot); [-1, 10])
\end{aligned}
\tag{15}
$$

The prior on the hyperparameter which captures the strength of a subject's prior beliefs (n_0) is a uniform distribution on $\log(n_0)$ with support from -1 (prior knowledge is equivalent to less than the observation of a single sequence) to 10 (prior knowledge corresponds to more than 100 times as many sequences as presented in the experiment). The same prior was used for the logarithm of the expected number of repetitions (-1: almost every tone will be followed by a different tone, 10: the same tone will be repeated throughout the entire experiment) and for the log-precision of sensory evidence (-1: σ is more than 10 times as large as the differences between two musical notes, 10: σ is less than one tenth of the difference between two musical notes).

4. Families of response models formalizing prediction error, model adjustment, and novelty detection

Prediction error hypothesis

Here, prediction errors were defined as the perceived value minus the value predicted from the past and weighted by the precision of its conditional probability density given its parents. In the case of the sensory input (u), the prediction was the mode of $p(u_t \mid \mathbf{z_{1:t\text{-}1}} = \mathbf{x_{z,2:t}}, \vartheta = \mathbf{x}_{\vartheta,\mathbf{t}})$ and the precision was the inverse variance of $p(u_t \mid z_t)$:

$$
\mathbf{L_5(t,:)} = \left(\sigma^{-2} \cdot \left(u_t - \sum \mathbf{x_{\omega,t}}(j) \cdot \mathbf{T}_{j,x_{z,t}}(x_{z,2:t}; x_{\eta,t}, x_{a,t}) \right) \quad 1 \right)
\tag{16}
$$

In the case of the hidden tone category ($\mathbf{z}$), the prediction was the mode of $p(z_t \mid \mathbf{z_{1:t\text{-}1}} = \mathbf{x_{z,2:t}}, \eta = x_{\eta,t}, \mathbf{a} = \mathbf{x_{a,t}})$ and the precision was its inverse variance.

$$\mathbf{L}_6(\mathbf{t},:) = \left(\frac{1}{\mathrm{Var}[Z_t \mid x_{z,1:t}, x_{\eta,t}, x_{a,t})]} \cdot \left(1 - \mathbf{T}_{x_{z,t}, x_{z,t+1}}(\mathbf{x}_{\mathbf{z},2:\mathbf{t}}; x_{\eta,t}, \mathbf{x}_{\mathbf{a},\mathbf{t}})\right) \quad 1 \right)$$
$$\mathrm{Var}[Z_t \mid \mathbf{z}_{\mathbf{1:t-1}} = \mathbf{x}_{\mathbf{z},2:\mathbf{t}}, \eta = x_{\eta,t}, \mathbf{a} = \mathbf{x}_{\mathbf{a},\mathbf{t}}] = \sum \mathbf{T}_{x_{z,t},k} \cdot (1 - \mathbf{T}_{x_{z,t},k}) \quad (17)$$

Novelty detection hypothesis

This hypothesis assumes that neuronal activity encodes conditional surprise (more formally surprisal) about the state of the world. The conditional surprisals can be interpreted as the observer's approximation to the stimulus-bound surprise, indexing novel events. We defined the conditional surprisal of an event (e.g., hearing a 500 Hz tone) as the negative logarithm of the conditional probability of that event under the observer's probabilistic prediction. We assumed that the observer's probabilistic prediction of the sensory input is a Gaussian with variance σ^2 centered on the mode of $p(u_t \mid \mathbf{z}_{\mathbf{1:t-1}} = \mathbf{x}_{\mathbf{z},2:\mathbf{t}}, \vartheta = \mathbf{x}_{\vartheta,\mathbf{t}})$. The conditional surprisals can be expressed in terms of prediction errors and precisions. Specifically, for a Gaussian density the conditional surprisal is a linear function of the precision weighted squared prediction error. Therefore, the surprisal about the sensory input u_t can be written as $-\ln \mathrm{p}\left(u_t | \mathrm{E}[Z_t | \mathrm{x}_{:,:t}], \sigma^2\right) = \frac{1}{2}\ln(2\pi\sigma^2) + \frac{1}{2\sigma^2}\left(u_t - \mathrm{E}[\omega_{Z_t} | \mathrm{x}_{:,:t}]\right)^2$, where the expected tone frequency is $\mathrm{E}[\omega_{Z_t} | \mathrm{x}_{:,:t}] = \sum_j \mathbf{x}_{\boldsymbol{\omega},\mathbf{t}}(j) \cdot \mathbf{T}_{x_{z,t},j}(x_{z,1:t}, x_{\eta,t}, x_{a,t})$:

$$\mathbf{L}_7(\mathbf{t},:) = \left(\frac{1}{2}\ln(2\pi\sigma^2) + \frac{1}{2\sigma^2}\left(u_t - \sum_j \mathbf{x}_{\boldsymbol{\omega},\mathbf{t}}(j) \cdot \mathbf{T}_{x_{z,t},j}(x_{z,1:t}, x_{\eta,t}, x_{a,t})\right)^2 \quad 1 \right) \quad (18)$$

Similarly, in the case of the category of the next tone, the probabilistic prediction was assumed to be $p(z_t \mid \mathbf{z}_{\mathbf{1:t-1}} = \mathbf{x}_{\mathbf{z},2:\mathbf{t}}, \eta = x_{\eta,t}, \mathbf{a} = \mathbf{x}_{\mathbf{a},\mathbf{t}})$.

$$\mathbf{L}_8(\mathbf{t},:) = \left(-\ln \mathbf{T}_{k,i}(\mathbf{x}_{\mathbf{z},2:\mathbf{t}}; x_{\eta,t}, x_{a,t}) \quad 1 \right);\; k = x_{z,t}, i = x_{z,t+1} \quad (19)$$

Model adjustment hypothesis

As described in the main text, the model adjustment hypothesis assumes that the mismatch negativity reflects adjustments to the estimates of the parameters of the mental model. These parameters include the mean log-frequencies of the ten categories ($\omega_1, \cdots, \omega_{10}$), the expected number of repetitions (η) and the conditional change probabilities ($\mathbf{a}$). Specifically, the response models link the MMN to the updates of the mean log-frequency of the category of the current tone, the expected number of repetitions and the conditional probability of the observed transition. Furthermore, the effect of these updates on the MMN may or may not depend on the sign of the change. This implies a factorial structure of $3 \times 2 = 6$ response models.

$$\mathbf{L_9(t,:)} = \left(\mathbf{x_{\omega,t+1}}(x_{z,t+1}) - \mathbf{x_{\omega,t}}(x_{z,t+1}) \quad 1\right) \quad \mathbf{L_{12}}(t,:) = \left(\left|\mathbf{x_{\omega,t+1}}(x_{z,t+1}) - \mathbf{x_{\omega,t}}(x_{z,t+1})\right| \quad 1\right)$$

$$\mathbf{L_{10}(t,:)} = \left(x_{\eta,t+1} - x_{\eta,t} \quad 1\right) \qquad \mathbf{L_{13}(t,:)} = \left(\left|x_{\eta,t+1} - x_{\eta,t}\right| \quad 1\right) \tag{20}$$

$$\mathbf{L_{11}(t,:)} = \left(\mathbf{1}(x_{z,t+1} \neq x_{z,t}) \cdot \left(\mathbf{x_{a,t+1}}\left(x_{z,t+1}, x_{z,t}\right) - \mathbf{x_{a,t}}\left(x_{z,t+1}, x_{z,t}\right)\right) \quad 1\right)$$

The predictor variable of response model number 11 is the change in the posterior expectation of the conditional probability of the observed transition, if a transition has been inferred, and zero else. Since its value is never negative, its absolute value would yield the same prediction and is therefore not used for an additional response model. This is why there is only five rather than six response models formalizing the model adjustment hypothesis.

5. Approximation of the log-model evidence by sampling

Bayesian linear regression model with uninformative conjugate priors

The ith model's predictors ($\mathbf{L_i}$) were mapped to the measured MMN amplitudes via a multivariate Bayesian linear regression model with conjugate priors. This section describes—in a generic way—how statistical inference was conducted with this model. The data $\mathbf{Y} = \left(\mathbf{y}^{(1)}_{\mathbf{1,\ldots,n_d}} \quad \cdots \quad \mathbf{y}^{(\kappa)}_{\mathbf{1,\ldots,n_d}}\right)$ consist of K dependent variables $\mathbf{y}^{(1)}, \cdots, \mathbf{y}^{(\mathbf{K})}$ comprising n_d observations each. The likelihood of the MMN amplitudes recorded at a single electrode ($\mathbf{y}^{(k)}$) is:

$$p(\mathbf{y}^{(\mathbf{k})} \mid \mathbf{L}_i, \boldsymbol{\beta}^{(\mathbf{k})}, \sigma_\varepsilon^2) = N(\mathbf{y}^{(\mathbf{k})}; \mathbf{X_i}(\boldsymbol{\theta})\boldsymbol{\beta}^{(\mathbf{k})}, \sigma_{\varepsilon,k}^2 \cdot \mathbf{I}) \tag{21}$$

Here, $\mathbf{X_i}(\boldsymbol{\theta})$ denotes the design matrix that was created by replacing the non-constant columns of $\mathbf{L_i}$ by their z-transforms; its dependence on the parameters $\boldsymbol{\theta}$ of the evolution function is made explicit, because it will be important below. $\boldsymbol{\beta}^{(\mathbf{k})}$ are the regression coefficients for the kth electrode, and $\sigma_{\varepsilon,k}$ is the standard deviations its measurement error. The priors on the parameters $\boldsymbol{\beta}$ and σ_ε were chosen to be uninformative and conjugate to this likelihood function.

$$p\left(\boldsymbol{\beta}^{(\mathbf{k})}, \sigma_{\varepsilon,k}^2\right) = N\left(\boldsymbol{\beta}^{(\mathbf{k})}; \boldsymbol{\mu}_{\boldsymbol{\beta}}, \boldsymbol{\Lambda}_\mathbf{0}^{-1} \cdot \sigma_{\varepsilon,k}^2\right) \cdot \mathrm{InvGamma}(\sigma_{\varepsilon,k}^2; a_0, b_0) \tag{22}$$

Here, $\mathrm{InvGamma}\left(\sigma^2; a, b\right) = \frac{b^a \left(\sigma^2\right)^{-a-1}}{\Gamma(a)} \cdot \exp(-b/\sigma^2)$. The values $a_0 = 2.01$ and $b_0 = 1.01$ were chosen to make the inverse Gamma prior uninformative with $E\left[\sigma_\varepsilon^2\right] = \frac{b}{a-1} = 1$ and $\mathrm{Var}\left[\sigma_\varepsilon^2\right] = \frac{b^2}{(a-1)^2(a-2)} = 100$, c.f. [3]. The mean of the Gaussian prior on $\boldsymbol{\beta}^{(\mathbf{k})}$ was chosen such that it contains no information about the direction of the effect, i.e. $\boldsymbol{\mu}_\mathbf{0} = 0 \cdot \mathbb{1}_l$, where l is the number of

regressors. The precision matrix $\mathbf{\Lambda_0} = \kappa \cdot \mathbf{I}_{l \times l}$ of the prior on $\boldsymbol{\beta}$ was chosen such that $P\left(-80 \leq \hat{y}_t^{(k)} \leq 80\right) = 0.95$ for each trial t, where $\hat{\mathbf{y}}^{(\mathbf{k})} = \mathbf{X_i}(\boldsymbol{\theta}) \cdot \boldsymbol{\beta}^{(\mathbf{k})}$ is the predicted amplitude. Since we standardized the regressors such that they have mean zero and variance one, predictive amplitudes are normally distributed with mean zero and variance $l \cdot \sigma_\beta^2$. Therefore, we set $\sigma_\beta = 80/(1.96\sqrt{l})$.

Computation of the log-model evidence

The chosen Bayesian linear regression model makes it easy to compute the log-model evidence. First, $p(\mathbf{y} \mid \mathbf{X}_i(\boldsymbol{\theta}), M_i)$ factorizes into the marginal likelihoods of the MMN amplitudes at the individual electrodes, i.e. $p(\mathbf{y} \mid \mathbf{X}_i(\boldsymbol{\theta}), M_i) = \prod_k p(\mathbf{y}^{(\mathbf{k})} \mid \mathbf{X}_i(\boldsymbol{\theta}), M_i)$. Second, we have chosen conjugate priors. Therefore the marginal likelihood $p(\mathbf{y_e} \mid \mathbf{X}(\boldsymbol{\theta}), m)$ of the MMN amplitudes measured at each individual electrode can be computed analytically. Specifically, the marginal likelihood of the amplitudes recorded by a single electrode can be written as follows [4], where the variables with subscript n refer to the parameters of the posterior:

$$p\left(\mathbf{y}^{(\mathbf{k})} | \mathbf{X}_i(\boldsymbol{\theta}), M_i\right) = \frac{1}{(2\pi)^{\frac{n}{2}}} \sqrt{\frac{\det(\mathbf{\Lambda}_0)}{\det(\mathbf{\Lambda}_n)}} \cdot \frac{b_0^{a_0}}{b_n^{a_n}} \cdot \frac{\Gamma(a_n)}{\Gamma(a_0)} \tag{23}$$

Since the change detection models have no free parameters $\boldsymbol{\theta}$, their log-model evidence is given by equation (23). For the adaptation model and the Bayesian models, the log model evidence $p(\mathbf{y} \mid M_i)$ is the integral $\int p(\mathbf{y} \mid \mathbf{X}_i(\boldsymbol{\theta}), \boldsymbol{\beta}, \sigma, M_i) \cdot p(\boldsymbol{\theta}, \boldsymbol{\beta}, \sigma_\varepsilon^2 \mid M_i) d\boldsymbol{\beta} d\sigma_\varepsilon^2 d\boldsymbol{\theta}$. This integral can be rewritten as $\int p(\mathbf{y} \mid \mathbf{X}_i(\boldsymbol{\theta}), M_i) d\boldsymbol{\theta}$ and the restricted likelihood $p(\mathbf{y} \mid \mathbf{X}_i(\boldsymbol{\theta}), M_i)$ can be computed according to equation (23). The integral with respect to the parameters $\boldsymbol{\theta}$ of the computational models, however, cannot be computed analytically and thus has to be approximated. For the adaptation model (M_4), we approximated this integral by Monte-Carlo integration. Specifically, we approximate the evidence of adaptation model by the average likelihood of $100'000$ parameter vectors $\boldsymbol{\theta}^{(k)}$ sampled from its prior (p_4).

$$\begin{aligned} p(\mathbf{y} \mid M_4) &= \int p(\mathbf{y} \mid \mathbf{X}_4(\boldsymbol{\theta}), M_4) \cdot p_4(\boldsymbol{\theta}) d\boldsymbol{\theta} \\ &\approx \frac{1}{n} \sum_{k=1}^{n} p(\mathbf{y} \mid \mathbf{X}_4(\boldsymbol{\theta}^{(k)}), M_4) \text{ with } \boldsymbol{\theta}^{(k)} \sim P_4 \end{aligned} \tag{24}$$

The log model evidence of the free-energy based models was approximated using a variant of Monte-Carlo integration known as importance sampling:

$$\forall M_i \in \mathrm{fam}_{\mathrm{FEP}} : p(\mathbf{y} \mid M_i) \approx \frac{1}{n}\sum_{k=1}^{n} p(\mathbf{y} \mid \mathbf{X}_i(\tilde{\boldsymbol{\theta}}_k), M_i) \cdot \frac{p_i(\tilde{\boldsymbol{\theta}}_k)}{q(\tilde{\boldsymbol{\theta}}_k)} \text{ with } \tilde{\boldsymbol{\theta}} \sim Q \tag{25}$$

Specifically, we have chosen the following proposal distribution (Q), because we expect it to resemble the joint distribution more closely than does the prior.

$$q(\boldsymbol{\theta}) = \mathrm{Exp}[n_0; \lambda_n = 1/1000] \cdot \mathrm{Exp}[\eta_0; \lambda_\eta = 1/1000] \cdot \mathrm{Uniform}(\log(\sigma^{-2}); [-1;10]) \tag{26}$$

Here, $\mathrm{Exp}[x;\lambda]$ denotes the exponential distribution with mean $1/\lambda$. Again, each model evidence estimate was based on 100'000 samples.

6. Neurobiological Implementation

In neurobiological terms, one could envisage the optimization described above to be implemented in a predictive coding circuit similar to the one proposed in [5]. Furthermore, one can speculate that the posterior expectations about variables at the two levels of the mental model m in Figure 3 are represented at different levels of the hierarchy of auditory cortex [6,7]. Based on what we know about auditory neurophysiology [8] and recent neuroimaging results about the neurophysiological mechanisms that generate the MMN [9] one might postulate that log-frequency ($\mathbf{u}$) is represented in primary auditory cortex (A1) and that the next higher level of the probabilistic mental model ($\mathbf{z}$) might be represented in a secondary auditory area within the superior temporal gyrus (STG). Furthermore, according to predictive coding under the free-energy principle [10] each of these areas should comprise representational units that cast predictions via top-down projections to lower auditory areas and error units that signal precision weighted prediction error to higher cortical areas. In the following two sections, we will use this framework to speculate how the brain might implement the two variational updates described above.

Neurobiological Implementation of Variational Update 1 (Perceptual Inference)

Auditory cortex could implement the free-energy minimization with respect to the posterior expectation of the hidden state z_t by using several populations of neurons that compute the free-energies associated with each possible mode of the approximate posterior on the hidden state ($x_{z,t+1}$). Since the equations for these free-energies (Eq. (9)) can be written in terms of predictions errors, the brain could represent them by the activity of prediction error units. Concretely, in each of the three cases the second term corresponds to the error in the prediction of the sensory input given a particular choice of the hidden tone category, cf. Eq. (16). Therefore, the brain might represent these terms by c populations of error units in primary auditory cortex that encode the precision weighted prediction errors associated with each of the c possible choices for the hidden category ($\xi_1^{(1)}, \cdots, \xi_c^{(1)}$). Similarly, the first terms correspond to the error in the prediction of the hidden state given all previous hidden states, if there were any, cf. Eq. (17). Such prediction errors may be encoded by error units in secondary auditory cortex for each possible value of the hidden category ($\xi_1^{(2)}, \cdots, \xi_c^{(2)}$). Together the error units in primary and secondary auditory cortex encode the free-energies associated with each possible choice for the mode of the approximate posterior on the hidden category (Eq. (9)).

Given a neuronal representations of these free-energies, secondary auditory cortex could infer the tone category by selecting the value for which the free-energy is lowest, c.f. Equation (27) .

$$x_{z,t+1} = \arg\min_z - \mathcal{F}(\xi_z^{(1)}, \xi_z^{(2)}) \tag{27}$$

Several neurophysiological implementations of this decision mechanism are conceivable (e.g., [5,10]). Importantly, the free-energies associated with the two options are linear polynomials of precision weighted squared prediction errors. These can be computed from the prediction errors signals from A1 ($\boldsymbol{\xi}_{1:c}^{(1)}$) and the activity of prediction error units in the same area ($\boldsymbol{\xi}_{1:c}^{(2)}$). Therefore, this area could employ one neural population for representing the free-energy of each option and use a winner-takes-all circuit to read out which of them is least active.

Neuronal Implementation of Variational Update 2 (Perceptual Learning)

In this section we refer to previously made arguments [5,10,11] that synaptic plasticity could implement the learning of the parameters ϑ in the brain's probabilistic mental model m according to the free-energy principle. These parameters determine how the brain predicts the variables at each level of the mental model m in Figure 3 given the variables at the next higher level. Top-down projections compute the predictions encoded by the representational units at each level and convey them to error units at the next lower level. The synaptic configurations at these projections embody the parameters. Therefore, perceptual learning might be instantiated by synaptic plasticity at top-down projections [5]. One can, at least in certain cases, derive differential equations that prescribe the gradient descents on the free-energy with respect to these parameters and interpret them in terms of synaptic plasticity [5,12]. That is, the expectation of the observer's belief about a parameter may be encoded by synaptic strengths and the associated precisions may be reflected by the capacity for plastic changes. The latter may be controlled by neuromodulators such as dopamine [11] or acetylcholine [13].

References

1. Dempster AP, Laird NM, Rubin DB (1977) Maximum Likelihood from Incomplete Data via the EM Algorithm. Journal of the Royal Statistical Society Series B (Methodological) 39: 1-38.
2. Daunizeau J, den Ouden H, Pessiglione M, Kiebel S, Stephan K, et al. (2010) Observing the Observer (I): Meta-Bayesian Models of Learning and Decision-Making. PLoS ONE 5: e15554.
3. Penny W, Trujillo-Barreto N, Friston K (2005) Bayesian fMRI time series analysis with spatial priors. NeuroImage 24: 350-362.
4. O'Hagan A (1994) Bayesian Inference: Halsted.
5. Friston K (2005) A theory of cortical responses. Philosophical transactions of the Royal Society of London Series B, Biological sciences 360: 815-836.
6. Romanski L, Averbeck B (2009) The Primate Cortical Auditory System and Neural Representation of Conspecific Vocalizations. Annual Review of Neuroscience 32: 315-346.
7. Friston K (2008) Hierarchical models in the brain. PLoS computational biology 4: e1000211.
8. Schnupp J, Nelken I, King A (2010) Auditory Neuroscience: Making Sense of Sound: The MIT Press.
9. Garrido M, Kilner J, Stephan K, Friston K (2009) The mismatch negativity: a review of underlying mechanisms. Clinical Neurophysiology 120: 453-463.

10. Friston K, Kiebel S (2009) Predictive coding under the free-energy principle. Philosophical transactions of the Royal Society of London Series B, Biological sciences 364: 1211-1221.
11. Friston K, Shiner T, FitzGerald T, Galea J, Adams R, et al. (2012) Dopamine, affordance and active inference. PLoS computational biology 8: e1002327.
12. Friston K (2003) Learning and inference in the brain. Neural Netw 16: 1325-1352.
13. Yu A, Dayan P (2005) Uncertainty, Neuromodulation, and Attention. Neuron 46: 681-692.
